# Supplementary figures and images for: Classes and continua of hippocampal CA1 inhibitory neurons revealed by single-cell transcriptomics
Source: PLoS Biol. 2018 Jun 18;16(6):e2006387. doi: 10.1371/journal.pbio.2006387 (PMC6029811; doi:10.1371/journal.pbio.2006387)

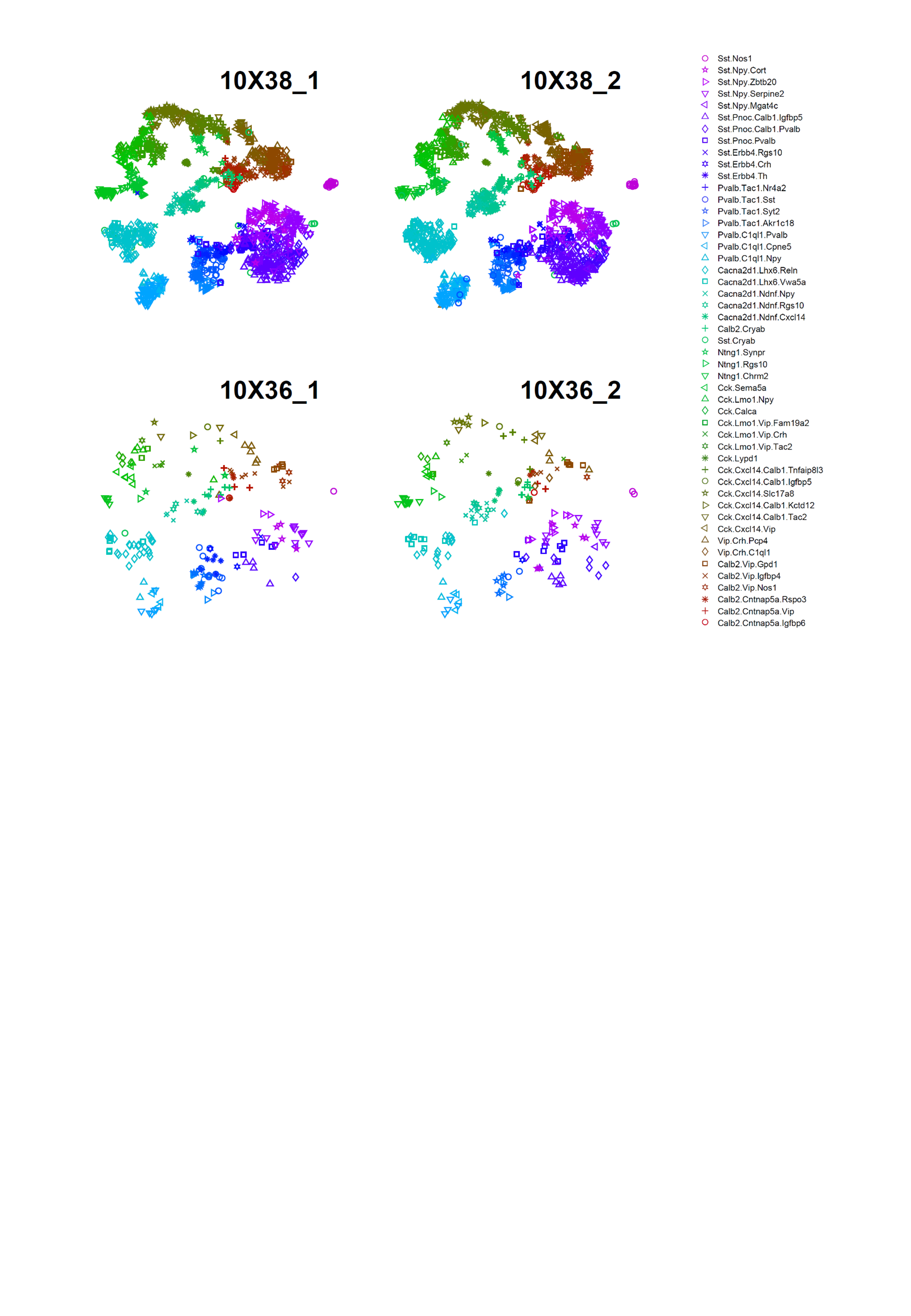

Supplement: S1 Fig — Each plot shows the location of all cells collected in a sample on the nbtSNE plots. Top row: mice of age p60; bottom row, age p27. Statistical testing of homogeneity of cluster IDs between mice yielded a weakly significant overall result (p = 3 × 10^−5^; χ2 test), but post hoc analysis (individual 2×2 contingency tables, Bonferroni corrected) did not identify any clusters with individually significant differences between mice. We suspect that the weakly significant difference might reflect slightly differential dissection of layers in the four samples. nbtSNE, negative binomial t-stochastic neighbor embedding. (TIF) [file pbio.2006387.s001.TIF]

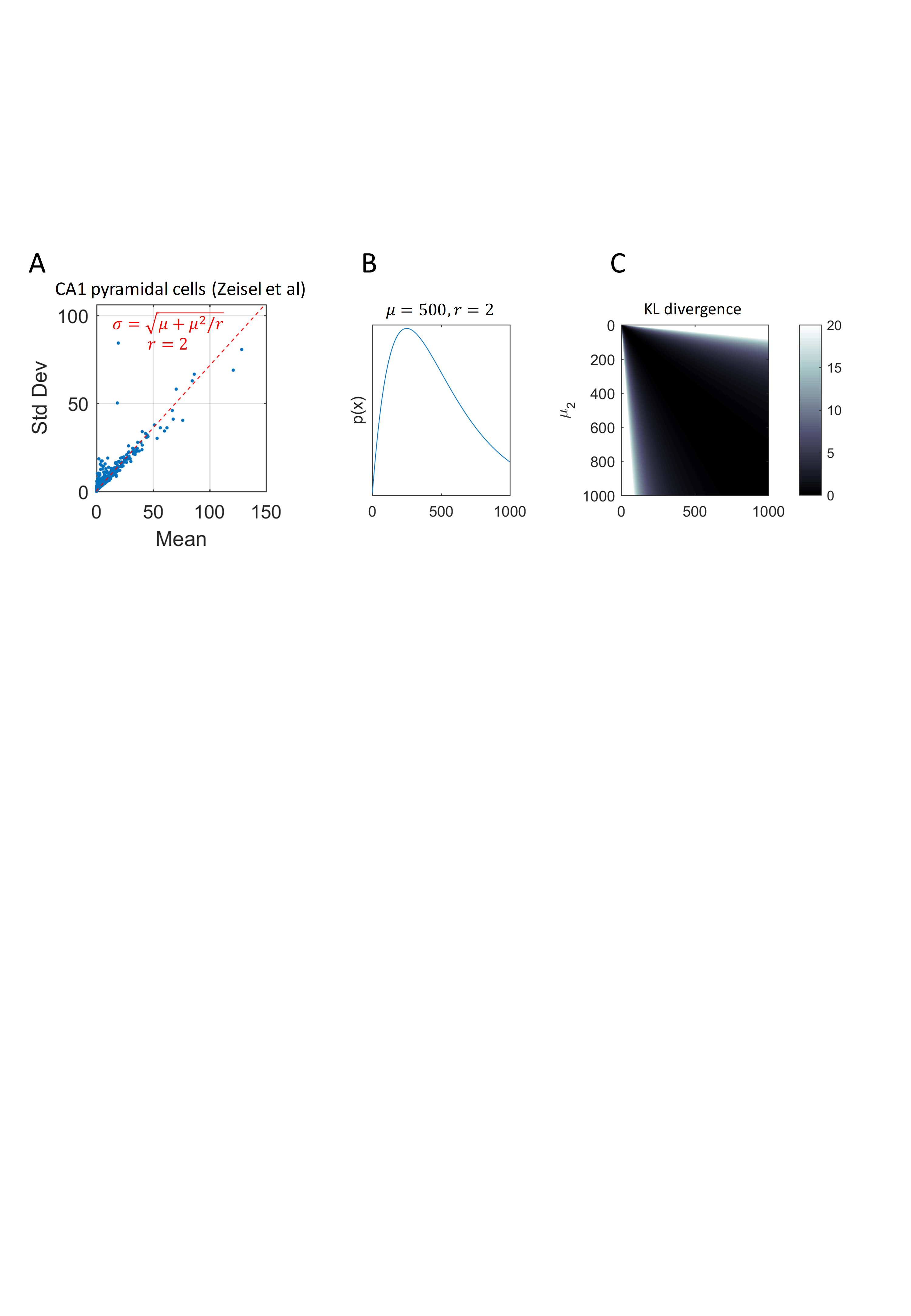

Supplement: S2 Fig — (A) Standard deviation versus mean expression in a population of CA1 pyramidal cells (Zeisel et al. 2015). Each point represents a single gene. Red curve shows prediction of negative binomial distribution with r = 2. (B) Example negative binomial probability distribution. Low probability is assigned to very small counts, but for larger counts, there is no strong dependence on precise count value. (C) Symmetrized Kullback-Liebler divergence between two negative binomial distributions, as a function of their mean values. High values (indicating poor fit) are obtained when one mean is close to zero, and the other is large; when both means are far from zero, their precise values do not have a major effect. (TIF) [file pbio.2006387.s002.TIF]

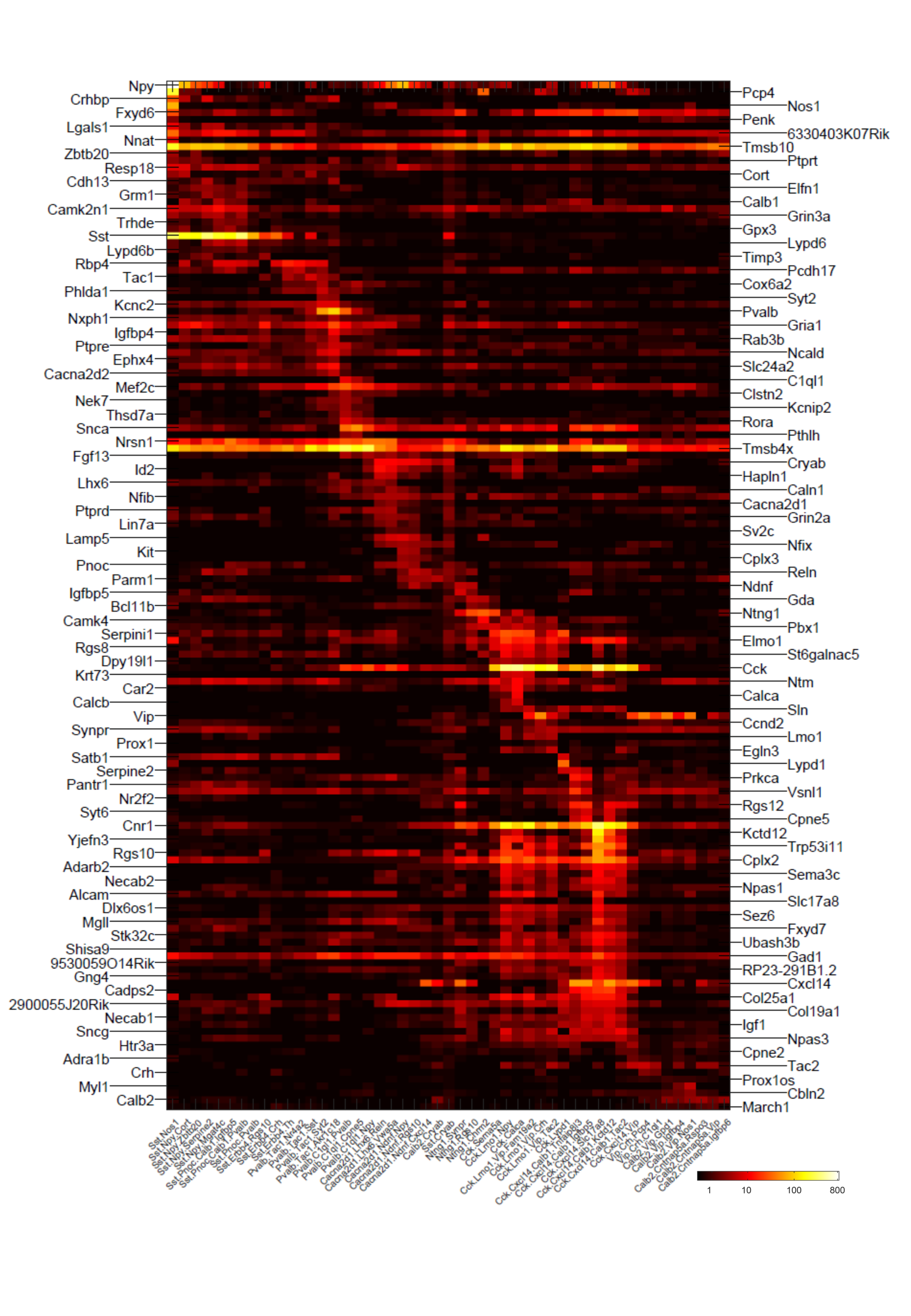

Supplement: S3 Fig — ProMMT, Probabilistic Mixture Modeling for Transcriptomics. (TIF) [file pbio.2006387.s003.TIF]

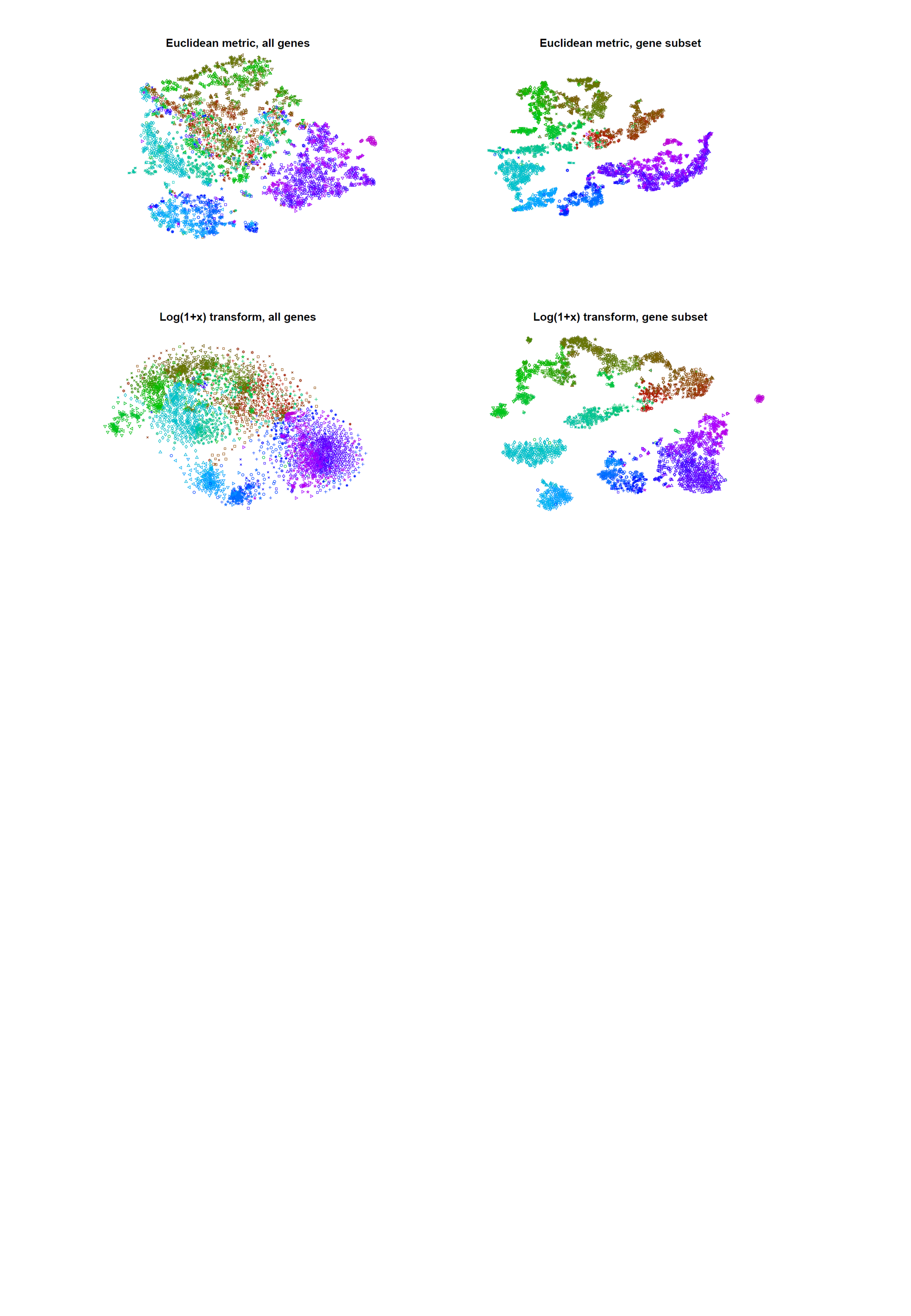

Supplement: S4 Fig — Left columns show results when all genes are used; right columns show results with the 150 genes selected by ProMMT. Top row shows a Euclidean metric; bottom row shows Euclidean metric after log(1+x) transformation. All methods were initialized from the same starting point as nbtSNE. Of the four methods, only the log(1+x) transformed data with gene subset gave comparable results to nbtSNE. This indicates that the primary effect of the negative binomial distribution is to downweight differences in expression between strongly expressed genes, similarly to the log(1+x) transformation, and that gene subsetting produces more interpretable results whether or not transformation is used. nbtSNE, negative binomial t-stochastic neighbor embedding; ProMMT, Probabilistic Mixture Modeling for Transcriptomics; tSNE, t-stochastic neighbor embedding. (TIF) [file pbio.2006387.s004.TIF]

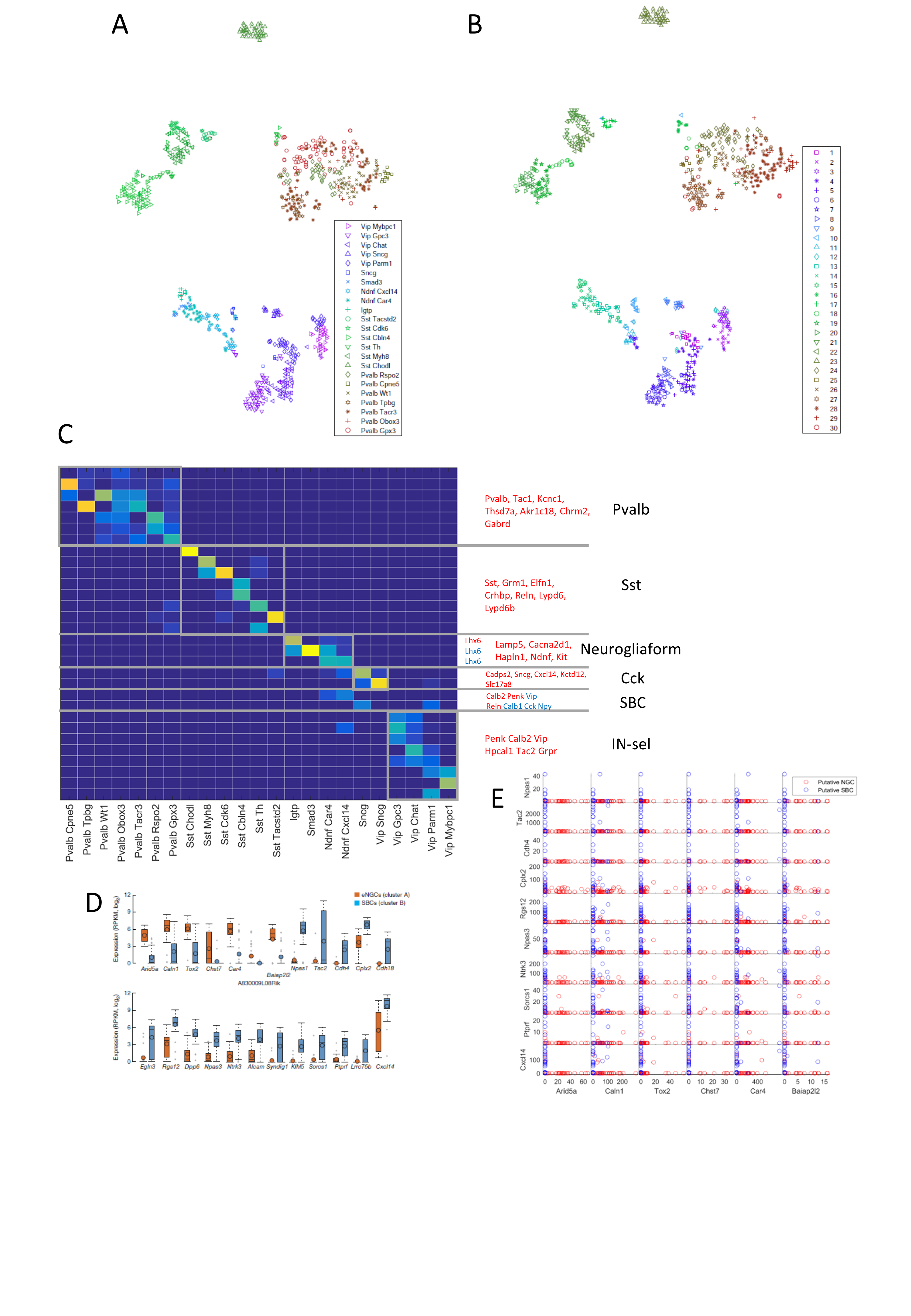

Supplement: S5 Fig — (A) nbtSNE algorithm applied to 761 interneurons of mouse V1, from Tasic and colleagues (2016). Symbols indicate 23 clusters assigned by Tasic and colleagues. (B) Same data, with symbols representing 30 clusters assigned by ProMMT algorithm. (C) Confusion matrix relating cluster assignments made by the two algorithms. Right; cell classes identified with ProMMT clusters (black), and genes used to make the identification (red: expressed, blue: not expressed). (D) Reprint of figure from Cadwell and colleagues (2016) showing expression of selected genes in layer 1 SBCs and eNGCs. (E) Scatterplot matrix showing expression of these genes in Tasic and colleagues’ data support the identification of SBCs made by ProMMT algorithm. eNGC, elongated neurogliaform cell; nbtSNE, negative binomial t-stochastic neighbor embedding; ProMMT, Probabilistic Mixture Modeling for Transcriptomics; SBC, single-bouquet cell. (TIF) [file pbio.2006387.s005.TIF]

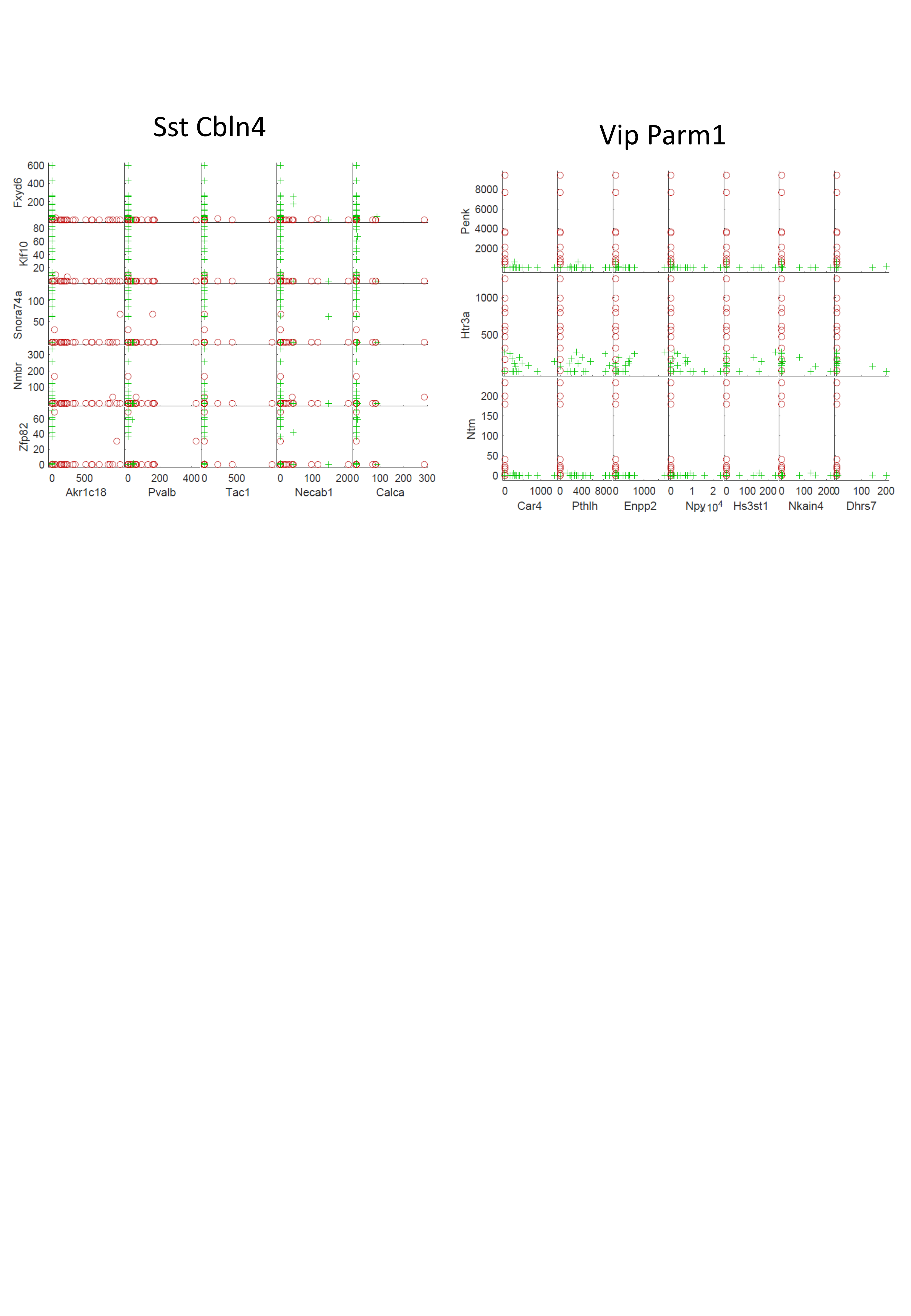

Supplement: S6 Fig — Left and right panels show scatterplot matrices for sets of genes with near-exclusive expression in further subdivisions of the Vip Parm1 and Sst Cbln4 clusters. Red and green points indicate which subcluster the cell was placed in by the ProMMT algorithm. ProMMT, Probabilistic Mixture Modeling for Transcriptomics. (TIF) [file pbio.2006387.s006.TIF]

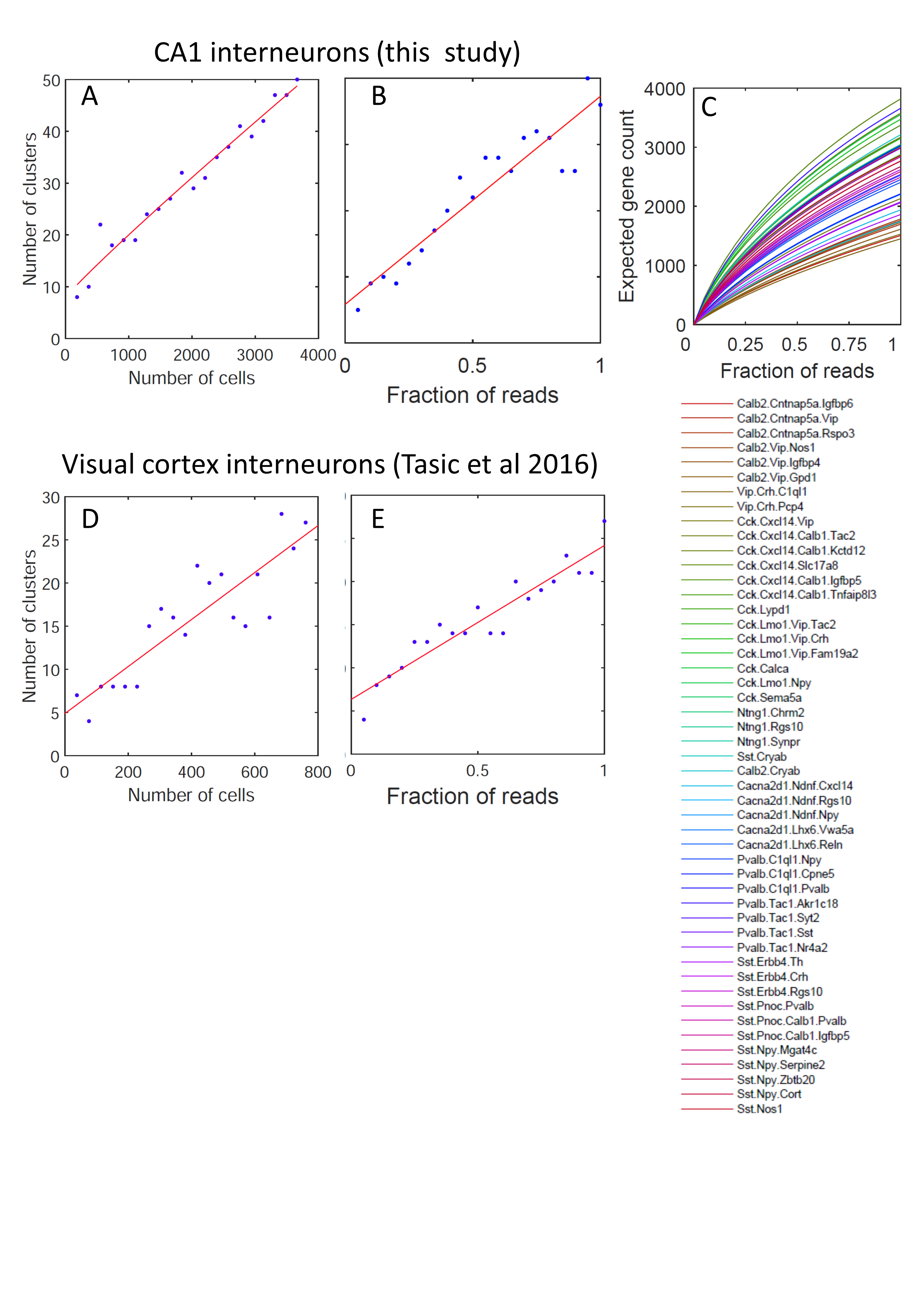

Supplement: S7 Fig — (A) To investigate how the number of detected clusters might change with the number of cells analyzed, we reclustered random subsets of different numbers of cells. The number of clusters identified increased with cell count. (B) To investigate how the number of detected clusters might change with read depth, we resampled reads independently for each cell and gene, following a binomial distribution with probability between 0 and 1. Again, cluster count increased linearly with read depth; although a marginally sublinear trend was potentially visible, this was not statistically significant (p > 0.05, power-law regression). (C) Expected gene count (i.e., mean number of genes with expression >0, averaged over cells in a class), computed as a function of the binomial probability. Color scheme indicated below. (D,E) Similar analysis as (A,B) for the data of Tasic and colleagues (2016). (TIF) [file pbio.2006387.s007.TIF]

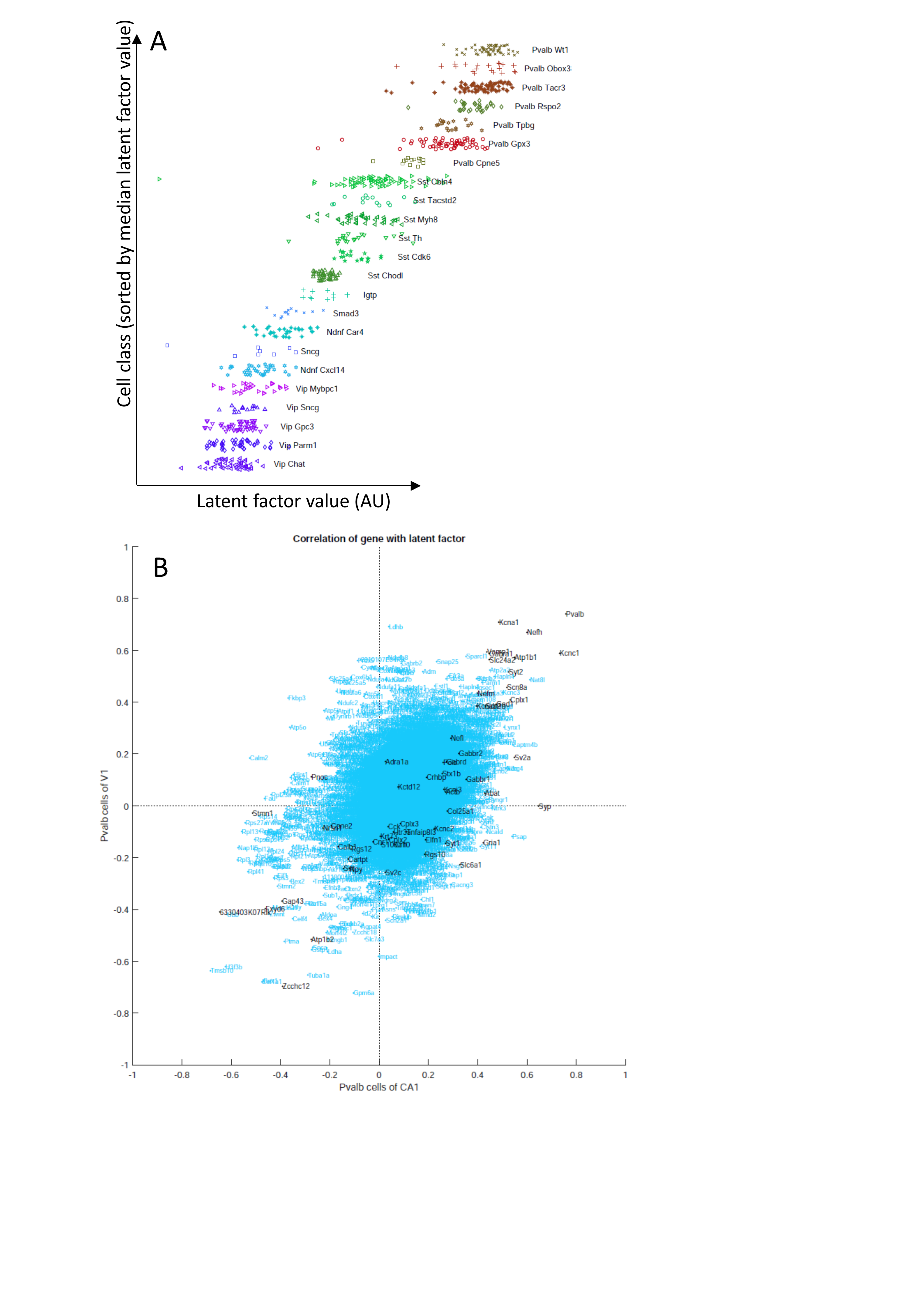

Supplement: S8 Fig — (A) Mean latent factor values differ between cell classes (cf. Fig 6A). Each point represents a cell; x-axis shows latent factor value; y-axis shows original cluster assignments. (B) Correlations of genes with the latent factor for isocortical Pvalb cells (y-axis) are similar to those of their CA1 counterparts (x-axis; cf. Fig 6D). (TIF) [file pbio.2006387.s008.TIF]

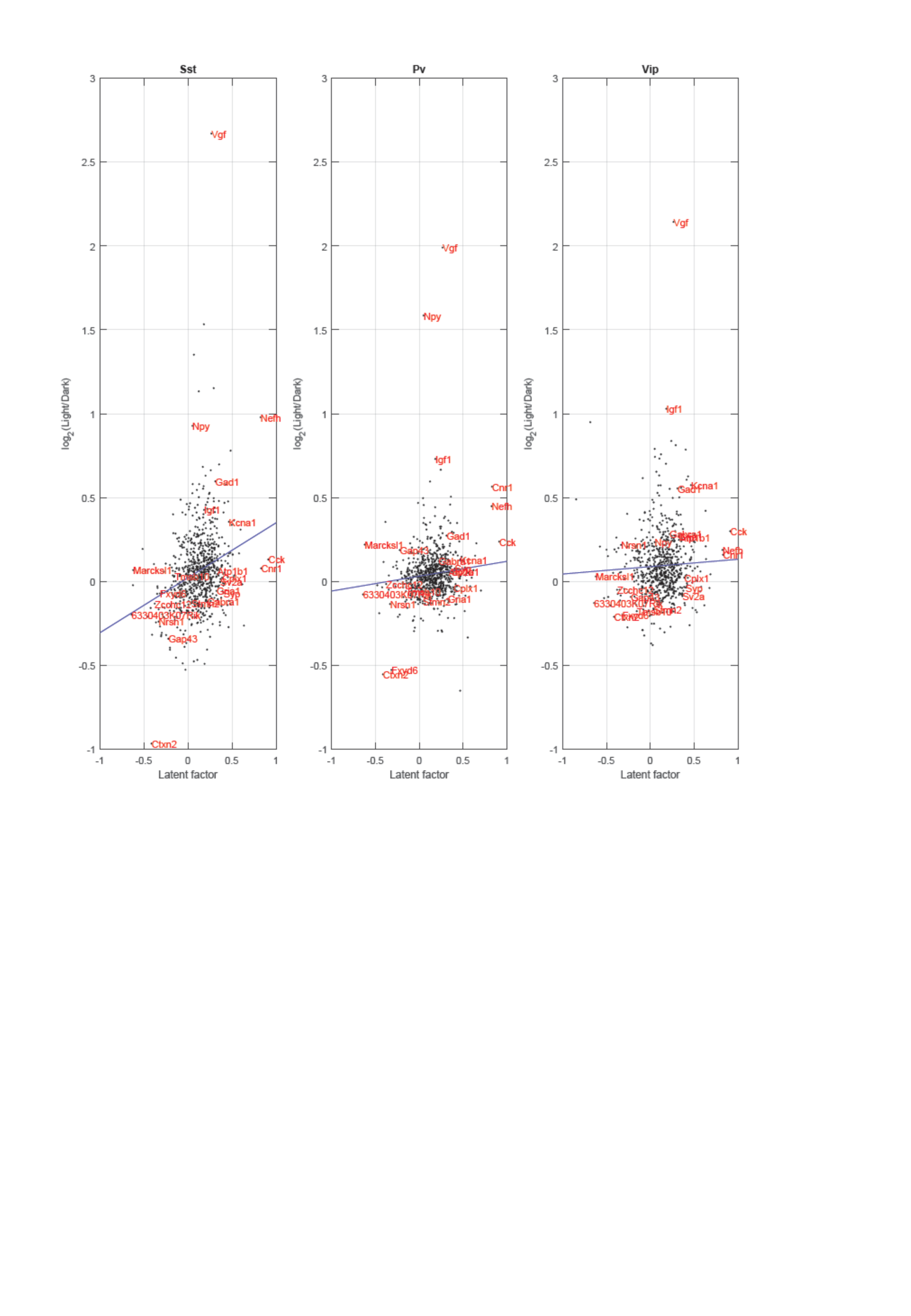

Supplement: S9 Fig — Each panel represents a cell type analyzed by Mardinly and colleagues (2016). Within each panel, every point represents a gene, and the y-axis value shows the log ratio of its expression level after 7.5 h of light exposure, compared to dark housing, in the corresponding subtype of visual cortical interneurons. The x-axis value shows that gene’s latent factor weighting as determined from our CA1 data. Blue line shows linear regression fit, which was strongest for Sst neurons (r = 0.25; p < 10−12), weaker but significant for Pvalb neurons (r = 0.11; p < 0.002), and insignificant for Vip neurons (r = 0.05, p = 0.17). Only genes of strong mean expression were analyzed (>5,000 normalized reads). A small number of example genes of particular interest are highlighted with red text. (TIF) [file pbio.2006387.s009.TIF]

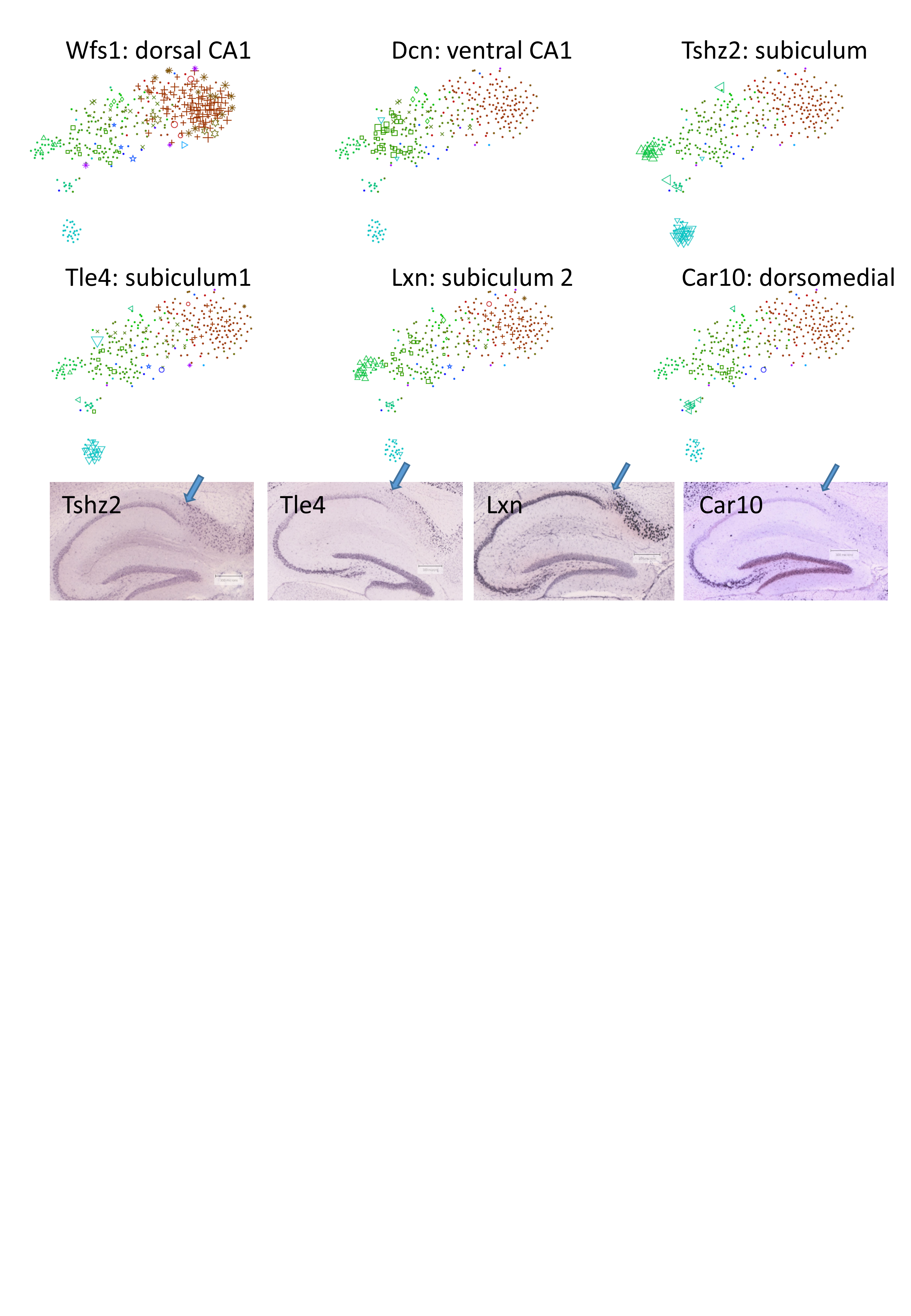

Supplement: S10 Fig — The main group of cells exhibited a continuous gradation of gene expression, consistent with previous reports of graded expression of genes such as Wfs1 and Dcn between dorsal and ventral CA1. No cells were identified as CA2 or CA3, as we did not detect populations consistently expressing genes such as Cacng5, Sostdc1, S100b, Ccdc3, Iyd, or Coch. However, three small clusters together containing 62 of the total 357 excitatory neurons were identified as either occurring at the dorsomedial lip of stratum oriens or subiculum, because of their expression of genes such as Tshz2, Tle4, Lxn, and Car10, whose Allen atlas expression patterns are shown at bottom. (TIF) [file pbio.2006387.s010.TIF]
