# Supplementary material for: Classes and continua of hippocampal CA1 inhibitory neurons revealed by single-cell transcriptomics
Source: PLoS Biol. 2018 Jun 18;16(6):e2006387. doi: 10.1371/journal.pbio.2006387 (PMC6029811; doi:10.1371/journal.pbio.2006387)
Supplement: S1 Text — (PDF) [file pbio.2006387.s013.pdf]

# Classes and continua of hippocampal CA1 inhibitory neurons revealed by single-cell transcriptomics: S1 Text

Kenneth D. Harris[1,2,*], Hannah Hochgerner[3], Nathan G. Skene[1,3], Lorenza Magno[4], Linda Katona[5], Carolina Bengtsson Gonzales[3], Peter Somogyi[5], Nicoletta Kessaris[4], Sten Linnarsson[3], Jens Hjerling-Leffler[3]

1 – UCL Institute of Neurology, London UK
2 – UCL Department of Neuroscience, Physiology and Pharmacology, London UK
3 - Division of Molecular Neurobiology, Department of Medical Biochemistry and Biophysics, Karolinska Institutet, Stockholm, Sweden
4 – UCL Wolfson Institute for Biomedical Research, London UK
5 – Department of Pharmacology, University of Oxford, Oxford UK
* Correspondence: kenneth.harris@ucl.ac.uk

## Detailed Rationale for Cell Type Identifications

Here, we describe in depth the identifications of our transcriptomic clusters with known cell classes. The evidence for these identifications comes from two main sources. Primarily, we have leveraged the very large body of knowledge derived from immunohistochemical analyses. Most, but not all, of these studies were performed in rat. Nevertheless, there are strong similarities between immunohistochemical analysis in mouse and rat [1], and we find only a few inconsistencies with this rat data, while also drawing heavily on a smaller but very informative set of gene expression studies in mouse [e.g. 2–7]. The second source of data is the Allen atlas [8]. We make some working assumptions throughout, for example that *Reln* labels cells whose axon projects to *stratum lacunosum-moleculare*, which is to our knowledge the case for all neuronal classes described so far [7,9].

The identifications of different cell classes are made with different levels of confidence, depending primarily on the detail with which the corresponding cell types have been molecularly characterized in the literature. To convey these confidence levels, we employ specific terminology. To say we "conclude" a particular identification, means a confidence level of 80% or more; "suggest" means confidence of 70%; "hypothesize" means 60%; while "speculate" means only 50%.

We refer to genes and proteins using HGNC conventions throughout, referring to mRNAs in italics (*Pvalb*), and proteins in capitals (PVALB). We abbreviate the hippompal layers as *so* (*stratum oriens*), *sp* (stratum pyramidale), *sr* (*stratum radiatum*), *slm* (*stratum lacunosum/moleculare*). When we say a group of cells is negative for a gene (abbreviated for example as *Npy-*), this means that its expression level is well below the overall average, but not necessarily that all cells in the group express exactly zero copies of the mRNA.

### *1. Cells expressing* Sst *and* Grm1*: O-LM, hippocamposeptal, and backprojection*

Neurons co-expressing SST and GRM1 are the dominant interneuron type in *so* [2,10–14], and have been divided into two primary

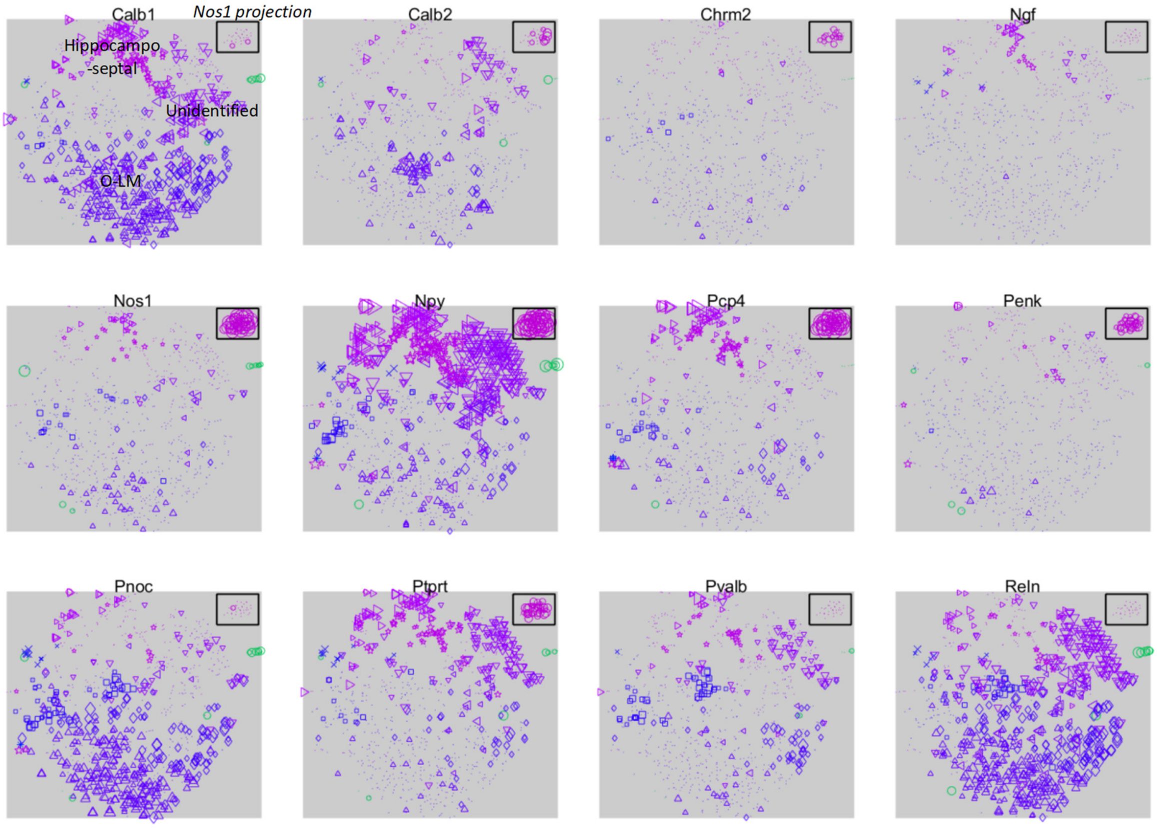


Fig **C1,10**. Expression patterns on continent 1 identify three major classes of *Sst/Grm1* expressing neurons and one potentially novel class. All cells express *Sst* and *Grm1* but not *Erbb4* (main text **Fig 3**). Hippocamposeptal cells are identified by expression of *Calb1* and *Ngf* but not of *Reln;* O-LM cells are identified by *Reln* but not *Npy;* cells expressing both *Npy* and *Reln* could not be identified with previously described cell types and are a suggested novel class. Inset: *Sst.Nos1* class (continent 10) has been moved from a distal location on the nbtSNE map. Symbols are as in main text Fig 2.

subgroups. The first subgroup, oriens-lacunosum/moleculare (O-LM) cells, are hippocampal homologs of isocortical Martinotti cells. O-LM cells have somas in *so* and axons that ramify in *slm* to inhibit pyramidal cell tuft dendrites. They express ELFN1, which gives them their characteristic facilitating excitatory inputs [15] and RELN, which we consider to be a marker of any cells projecting to *slm* [9,16]. However, few if any O-LM cells appear to express NPY (0/4 cells reconstructed cells were immunopositive in rat [16]). The second subgroup of SST/GRM1 cells project to the medial septum, and also receive reciprocal inputs from it [12,17,18]. It is likely that all hippocamposeptal cells also project retrohippocampally to the subiculum and possibly beyond, leading to their also being termed double projection cells [12]. Hippocamposeptal cells have somas located in *so*, often express CALB1 [12,19], around half express NPY [12], and a subset of them express NGF [20], with the NGF+ subset also more likely to express CALB1. From the many fully visualized examples it appears unlikely that O-LM cells can send collaterals to the septum, and most hippocamposeptal cells are distinct from O-LM cells, as their local axonal arbors ramify in *so* and *sr* while avoiding *slm* [12,18].

Our transcriptomic analysis revealed several clusters of cells co-expressing *Sst* and *Grm1* (n=901 cells total), located together in a large approximately circular continent toward the south-east of the nbtSNE map. Neurons within this group showed considerable diversity, although relatively low values of the isolation metric (averaging 25.5 bits/cell) suggesting that at least some of these clusters may represent points on a continuum. The most prominent distinction between these clusters concerned expression of *Npy*, with 3 clusters being *Npy-* and 4 clusters *Npy+*.

The three *Npy-* clusters matched the expected molecular signatures of O-LM cells (n=515 cells; **Fig C1**). They typically expressed *Reln* and *Elfn1* and sometimes *Pvalb*, which together with lack of *Npy* are considered O-LM signatures [7,9,16]. Some of these putative O-LM cells expressed *Calb2*, which has not been yet reported in O-LM neurons in rat, but has been found in a subset of isocortical *Sst* cells in mouse [21,22]; these *Calb2*-expressing neurons typically also expressed *Calb1.* In addition, these cells expressed several other markers including *Pnoc, Lypd6, Serpine2, Myh8, Mgat4c, Npas1* and *Npas3,* which we hypothesize may distinguish O-LM from hippocamposeptal cells. The three clusters of putative O-LM cells (*Sst.Pnoc.Pvalb*, *Sst.Pnoc.Calb1.Pvalb*, and *Sst.Pnoc.Calb1.Igfbp5*) showed a continuous gradation from cells expressing genes related to fast spiking activity (such as *Pvalb, Kcnc1, Gabra1*), to other genes including *Calb1* that may be associated with less rapid activity, which was mirrored in differences in the latent factor between these clusters.

Of the four *Npy*-expressing clusters (n=386 cells), two (*Sst.Npy.Cort* and *Sst.Npy.Zbtb20,* n=154 cells together) closely matched the expected expression profile of hippocamposeptal neurons. Indeed, in addition to *Sst, Grm1,* and *Npy,* these clusters expressed *Ngf* and *Calb1,* which together mark a subset of septally projecting cells [20]; furthermore they lacked *Reln*, suggesting that their axons did not reach *slm.* They were additional distinguished from putative O-LM cells by expression of number of other markers including *Pcp4, Ntrk3, Ptprt, Elmo1*, and *Vwc2.* Some cells in these clusters expressed *Htr3a*, but at low levels.

The remaining two *Npy*-positive clusters (*Sst.Npy.Mgat4c* and *Sst.Npy.Serpine2*) could not be as readily identified with previously described classes: although they expressed the expected markers of O-LM cells including *Reln*, their expression of *Npy*, albeit at relatively low levels, does not match previous reports of O-LM cells in rat [16]. Some of these cells expressed *Calb2*. We speculate they might constitute a novel class of *Npy*/*Sst*-expressing cells that have local axon collaterals in *slm*, such as a recently reported SST cell in the rat that differed from all previously reported neurons, also innervated the dentate gyrus and had a firing pattern different from O-LM and hippocamposeptal cells [23].

In summary, all clusters in the *Sst*+/*Grm1*+ group could be identified with previously described classes of O-LM, hippocamposeptal, and backprojection cells, with the exception of *Sst.Npy.Mgat4c* and *Sst.Npy.Serpine2*, which may constitute a novel class.

### *2. Cells expressing Pvalb and Tac1: basket and bistratified*

Three types of PVALB+ fast-spiking interneuron have been identified in the CA1 pyramidal layer: basket cells that target pyramidal somata; axo-axonic cells targeting axon initial segments, and bistratified cells targeting proximal dendrites [24]. These cells' fast-spiking phenotype arises from expression of a number of rapidly-acting ion channels, receptors, and synaptic proteins including

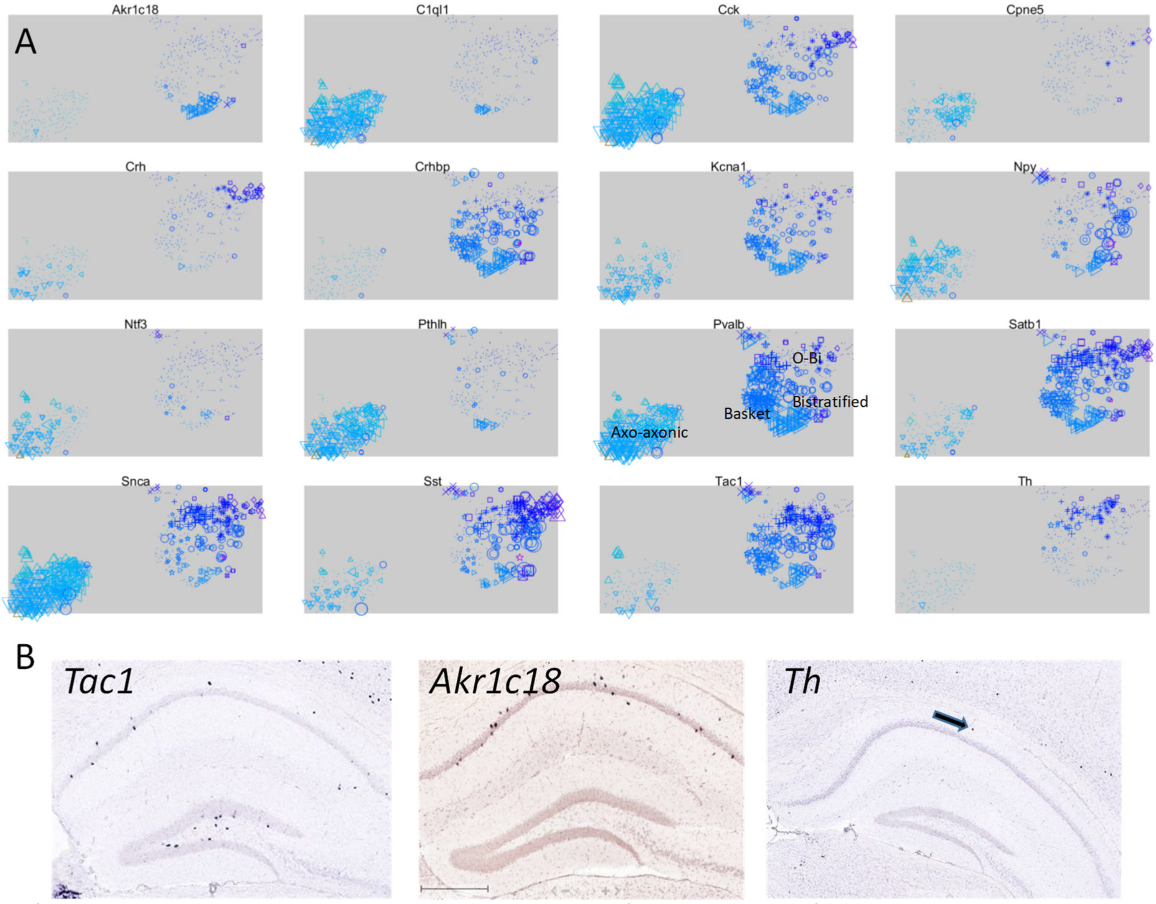


Fig **C2-3**. **A,** Expression patterns on continents 2 and 3 identify major classes of *Pvalb*-positive neurons. All cells express *Erbb4* (main text **Fig 3**). Continent 2, expressing *Satb1*, is identified as basket and bistratified cells. Expression of *Sst* and *Npy* identifies bistratified cells to the east, and lack of them identifies basket cells to the west. Lack of *Pvalb* identifies O-Bi cells to the north. Genes associated with fast-spiking phenotype (*Kcna1*, *Kcnc1*, *Pvalb*) are expressed most strongly towards the south of both basket and bistratified territory. Continent 3, lacking *Satb1*, is identified with axo-axonic cells. **B,** Identifications are confirmed by Allen atlas images showing expression of *Tac1* and *Akr1c18* in *sp* and *so* (as expected for basket and bistratified cells), and *Th* by rare cells in *so* (arrow), as expected for O-Bi cells. Symbols are as in main text Fig 2.

*Pvalb, Kcnc1, Kcna1, Gabra1, Gria1, Gria4, Cacna1a, Scn1a, Scn8a,* and *Syt2* [25]. All three classes express *Pvalb* but are distinct in other ways: bistratified cells express *Sst* and *Npy* [16], while axo-axonic cells are distinguished by their lack of *Satb1* [26]. In hippocampus and isocortex, expression of genes associated with fast-spiking activity (specifically *Kcna1* and *Pvalb*) are dynamically controlled by activity and learning [27,28]. Thus, while "delay-spiking" cells expressing *Kcna1* were once considered a separate cell type, it may be that this simply reflects a difference along a dynamically-regulated continuum of firing possibilities [27], specifically adapted to rapid spiking activity [25].

Continent 2 on the nbtSNE map contains cells that express genes associated with fast-spiking interneurons (e.g. *Pvalb, Kcnc1* and *Kcna1*) on its south and west sides, and cells expressing *Sst* and *Npy* in the east (**Fig C2-3**). This continent is connected to continent 1 by a thin "land bridge", but is distinguished from it by a lack of *Grm1,* and by expression of *Erbb4,* and *Tac1,* the neuropeptide precursor of substance

P and neurokinin A. The cells in this group express *Satb1*, and we therefore conclude they do not represent axo-axonic cells, but instead identify them with fast-spiking basket and bistratified cells, together with other related neurons.

Along the west side of continent 2 is a cluster *Pvalb.Tac1.Syt2,* that closely matches the expression profile of fast-spiking basket cells. Indeed, this cluster expressed *Pvalb, Kcnc1, Kcna1, Gabra1, Lhx6, Erbb4* and *Satb1* at high levels, but did not express *Sst, Npy, Grm1*, or *Vip*, while they expressed *Cck* and *Chrm2* at low levels, consistent with previous results [7,29]. An apparent continuum of expression was seen within this class, with cells to the south showing higher levels of the latent factor, corresponding to stronger expression genes associated with fast spiking (such as *Pvalb, Kcnc1, Kcna1, Scn1a, Scn1b, Syt1, Syt2*), GABA synthesis and vesicular release (*Gad1, Cplx1, Nsf, Sv2a, Syp, Dnm1),* fast synaptic inputs (*Gabra1, Gria1),* as well as the sodium-potassium exchanger *Atp1b1*, and several mitochondrial genes. Given that expression of both *Kcna1* and *Pvalb* are dynamically controlled by activity and learning [27,28], we suggest that this continuum reflects a continuum of activity-regulated genes, with cells toward the south expressing ion channels, receptors, exchangers, and buffers that enable a fast-spiking phenotype, together with the energy-production and synaptic genes required for rapid synaptic release. Several other genes were associated with this continuum, including a calcium sensor *Caln1,* the Calcium extruder *Slc24a2,* and neurofilament proteins (*Nefh, Nefl, Nefm),* whose association with a fast-spiking phenotype has to our knowledge not been investigated.

Further to the north, the *Pvalb.Tac1.Syt2* cluster abuts cluster *Pvalb.Tac1.Nr4a2*, that appears to be a continuation of the same continuum seen within *Pvalb.Tac1.Syt2.* Indeed, *Pvalb.Tac1.Nr4a2* cells did not express *Sst* or *Npy* (associated with bistratified cells) and contained many of the same markers as *Pvalb.Tac1.Syt2*, although those associated with fast-spiking or delay-spiking phenotypes (e.g. *Kcna1, Kcnc1, Syt2)* were expressed in still-lower amounts. Instead, a new set of genes were found toward this end of the continuum. Included in these genes were *Atp1b2* and *Fxyd6*, an alternative isoform and regulator of the sodium-potassium exchanger, which we speculate these might reflect a lower requirement for ion rebalancing due to lower firing rates. These cells also expressed higher levels of *Gabra2* compared to *Gabra1,* an isoform exhibiting slower kinetics [30], as well as several genes involved in regulation of actin (*Tmsb10, Gap43, Stmn2, Marcksl1)* as well as transcription factors (*Nr4a2, Zcchc12*), cell adhesion/recognition molecules (*Pcdh15*), and a gene of so-far unknown function (*6330403K07Rik*). The variation of many genes along this continuum, like the putative O-LM cell continuum in continent 1 (*Sst.Pnoc.Pvalb, Sst.Pnoc.Calb1.Pvalb*, and *Sst.Pnoc.Calb1.Igfbp5*), was strongly correlated with the hidden variable revealed by latent factor analysis (Main text **Fig 6C**).

We identified cells on the east side of this continent as bistratified cells, due to their expression of *Pvalb, Sst*, and *Npy*, but not *Elfn1* or *Grm1* [16]. These cells exhibited a very similar continuum of expression profiles to the putative basket cells, ranging from cells to the south that exhibited fast- and delay-spiking markers (cluster *Pvalb.Tac1.Akr1c18*) to those further north expressing many of the same novel markers associated with putatively slower-firing basket cells (cluster

*Pvalb.Tac1.Sst*). Intriguingly, neuropeptides (*Sst, Npy, Tac1, Cort*) were more strongly expressed in the putatively slower-spiking northern end of the continuum, while some additional genes not found in putative basket cells were expressed in the putatively fast-spiking south (e.g. *Akr1c18*, *Col25a1*, *Rgs8*, *Timp3*, *Thsd7a*).

To the north of this continent were 3 clusters of cells expressing *Sst* but not *Pvalb*, but were distinguished from putative O-LM and hippocamposeptal cells by lack of *Grm1, Reln,* or *Calb1,* and expression of *Tac1* and *Erbb4*. While identification of these cells with reports in the literature was less clear than for other cell types, their expression profile matches at least some of the diverse group described by Losonczy et al [31] as "Oriens-Bistratified" (O-Bi) cells. This identification is supported by the observation that oriens cells expressing the *Tac1* product substance P have a morphology consistent with that of bistratified cells [32]. Of the 3 clusters belonging to this group, *Sst.Erbb4.Rgs10* formed a rare but relatively distinct group (isolation metric 52.2 bits/cell) characterized by expression of *Npy, Rgs10* as well as genes putatively associated with faster-spiking activity (e.g. *Kcnc1*, *Col25a1*), located on a small island to the north-west of the main continent. The "land bridge" to the main *Sst* continent was occupied by cells expressing *Crh,* but lacking *Tac1* (*Sst.Erbb4.Crh*); expression of *Sema3c* was found in this group, unusually amongst *Sst* or *Pvalb* neurons. The final group (*Sst.Erbb4.Th*) showed an expression profile generally similar to bistratified cells but, in common with some of the putative slower-spiking basket cells, expressed *Th.* A dopaminergic or noradrenergic phenotype for these cells appeared unlikely, as they did not express the dopamine or noradrenaline transporters (*Slc6a2*, *Slc6a3*). These cells may form a hippocampal homolog of the *Th* expressing interneurons described by Tasic et al [21], which could be found amongst both *Sst* and *Pvalb* populations. Examination of the Allen Atlas [8] suggested a few *Th*-expressing cells were present in *so*, though much fewer than in deep isocortex [**Fig C2-3**].

### 3. Cells expressing Pvalb and C1ql1: axoaxonic

Axo-axonic cells are distinguished from other *Pvalb*-expressing interneurons by their lack of the transcription factor *Satb1* [26]. The cells of continent 3 matched this description, and also differed from putative basket and bistratified cells in many other respects. They lacked the substance P precursor *Tac1* but did express its receptor *Tacr1,* in contrast to the putative basket/bistratified cells that expressed *Tac1* but not *Tacr1.* A similar distinction between PVALB cells expressing either substance P or its receptor, but not both, has been observed immunohistologically in isocortex [33], which we suggest is homologous to our transcriptomic classes.

This group expressed *C1ql1,* a cell surface molecule involved in synapse formation [34], which was absent from putative basket cells and indeed only expressed in a few other groups. In addition, putative axo-axonic cells expressed *Snca*, *Cck*, *Pthlh*, *Ntf3* and *Trps1* much more strongly than putative basket and bistratified cells, but expressed *Rbp4* and *Crhbp* at much lower levels. This pattern of expression is similar to the isocortical "Pvalb Cpne5" class identified by Tasic et al [21], which were also identified as putative axo-axonic cells due to superficial laminar location.

The cells of continent 3 were more homogeneous than the others considered so far, and contained 3 clusters, which appeared to reflect a similar continuum of expression to that found in other classes. The cluster *Pvalb.C1ql1.Pvalb* (mean latent factor 1.06)

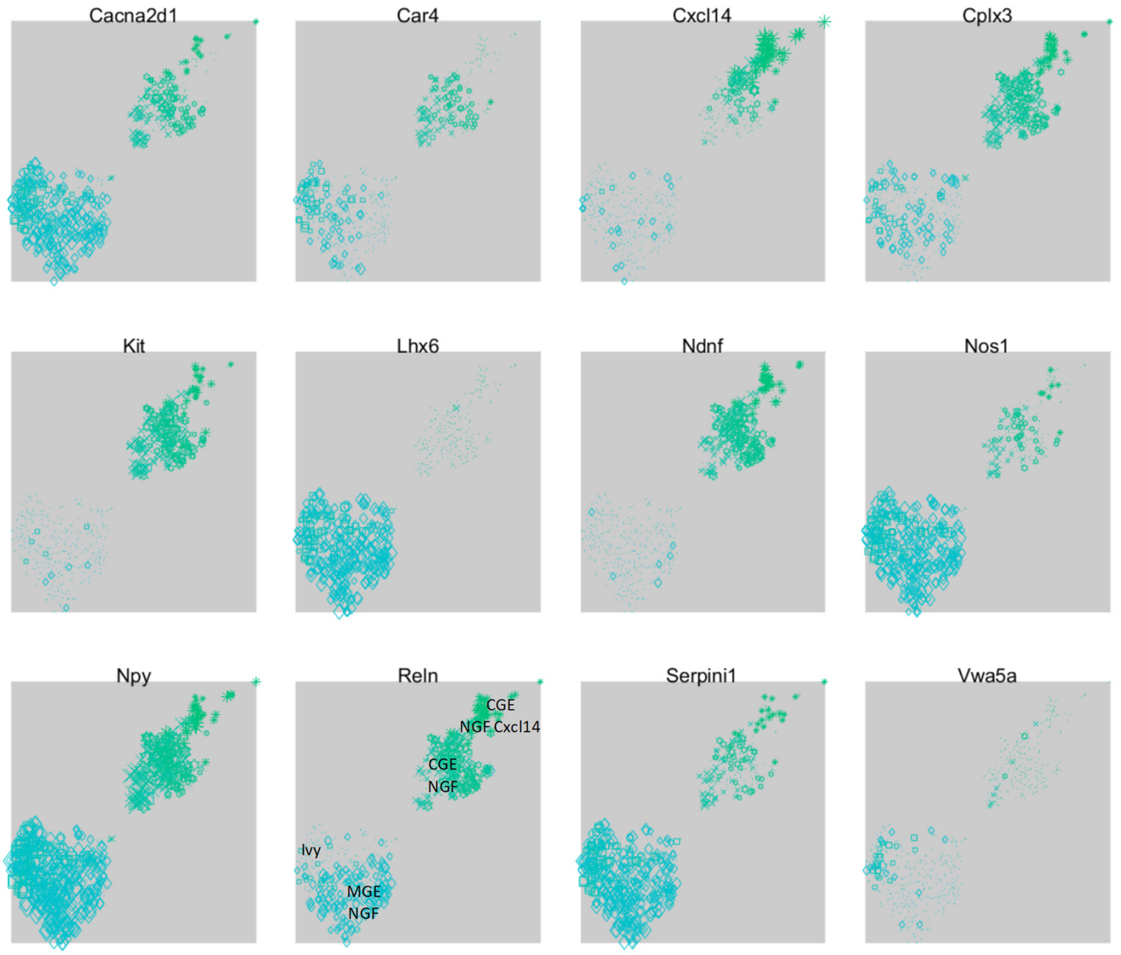


Fig **C4-5**. Neurons expressing *Cacna2d1* were identified as neurogliaform cells. Those expressing *Lhx6* and *Nos1* were identified as Ivy and MGE-neurogliaform cells, with Ivy cells distinguished by their lack of *Reln*. CGE-neurogliaform cells expressed distinct markers including *Kit* and *Ndnf*, and were separated into two groups by expression of either *Car4* or *Cxcl14*. Symbols are as in main text Fig 2.

showed stronger expression of genes associated faster-spiking phenotype (e.g. *Pvalb, Kcnc1, Kcna1, Caln1, Slc24a2*) while the remaining two clusters (mean latent factor 0.03 and 0.27) showed stronger expression of genes we associate with slower-firing activity (*6330403K07Rik, Fxyd6, Tmsb10, Zcchc12, Marcksl1*).

The two clusters of putative slower-firing axo-axonic cells differed through expression of several mainly novel genes. Cells in cluster *Pvalb.C1ql1.Cpne5* more strongly expressed *Cpne5, Pthlh, Pcdh10, Fam19a2* whereas cells in *Pvalb.C1ql1.Npy* more strongly expressed *Npy, Id2, Fabp5, Ntf3,* and *Cryab.* While physiological diversity amongst axo-axonic cells has been reported [35], a lack of previous molecular studies prevents identification of these subclasses with previously described types. Nevertheless, we note that Pascaul et al [36] described an overlap of *Ntf3* with *Pvalb* in a set of anatomically unidentified neurons located close to the CA1 pyramidal layer, which we hypothesize correspond to *Pvalb.C1ql1.Npy* cluster, the only one containing cells co-expressing these markers.

We note also that a very small group (7 cells) showed a distinct expression profile including additional expression of *Tac1*, resulting in an island location to the north of the main continent 3. These cells were not assigned a cluster, as their small number meant that the BIC penalty was not exceeded. Nevertheless, we speculate they might constitute a rare GABAergic class with hybrid features of basket and axo-axonic cells.

#### *4. Cells expressing Cacna2d1 and Lhx6: Ivy and MGE-neurogliaform*

Stratum lacunosum-moleculare contains a set of highly distinct GABAergic neurons known as neurogliaform cells, which are related to the ivy cells found in the strata oriens, pyramidale, and radiatum [6,37–39]. These cells have diverse developmental origins: Ivy cells and one subtype of neurogliaform cell that expresses *Nos1* have an origin in the medial ganglionic eminence (MGE); however a second type of neurogliaform cell originating in the caudal ganglionic eminence (CGE) expresses *Nos1* at most weakly [6]. Ivy and neurogliaform cells lack PVALB and SST, but express NPY, while neurogliaform but not ivy cells express RELN [38]. Neurogliaform and Ivy cells are also characterized by high levels of *Gad1*, *Gad2*, and *Gabra1* expression [6,38] and, at least in isocortex, by expression of GABRD [40].

We identified the fourth continent, located to the west of the expression map, with Ivy and MGE-derived neurogliaform cells (**Fig C4-5**). Indeed, these cells expressed *Npy* and *Nos1* but not *Pvalb* or *Sst*; they expressed *Lhx6* indicating a MGE origin; they showed amongst the strongest expression of *Gad1, Gad2, Gabra1,* and *Gabrd* of all cells in the database; and some, but not all of them expressed *Reln.*

These cells, together with a class of putative CGE-derived neurogliaform cells to their north-east, also expressed a set of novel markers, including *Cryab, Id2, Lamp5, Hapln1, Nfix, Bcl11b*, and *Cacna2d1.* The latter (a calcium channel auxiliary subunit that is a target of anti-epileptic drugs [41]) has an expression profile that is essentially unique to putative neurogliaform cells. In addition to *Lhx6* and *Nos1,* the putative MGE-derived cells were distinguished from putative CGE-derived neurogliaform cells by expression of genes including *Col25a1, Alcam,* and *Pkp4.* Surprisingly, *Actn2*, identified as a neurogliaform marker in rat [39], was not detected in these cells. We note however that this gene was found only at very low levels in any cells in our database, and further does not appear to have hippocampal expression in the Allen atlas. We therefore hypothesize the lack of this gene in our database may reflect a species difference between mouse and rat.

The cells of continent 4 were divided into two clusters, of which one (*Cacn1a2d1.Reln,* n=270 cells) contained *Reln* and was identified with MGE-derived neurogliaform cells, while the other (*Cacn1a2d1.Vwa5a,* n=86 cells) lacked *Reln* and was identified with Ivy cells. Evidence for a clear separation of these clusters, rather than a continuum, was not strong: the two clusters' isolation metrics were 31.7 and 13.1 bits/cell. Putative Ivy cells showed higher values of the latent factor (mean 0.25), showing greater expression of *Gabra1, Gabrd, Slc6a1, Caln1, Kcnip1,* while putative MGE-neurogliaform cells (mean latent factor -0.25) showed stronger expression of *Fxyd6* and *6330403K07Rik*. This result is consistent with the interpretation of the latent factor as reflecting proximal preference of inhibitory terminals on target cells, since Ivy cells have axons and boutons in the *sp* while neurogliaform cells target distal dendrites in *slm*. Putative Ivy cells also showed stronger expression of *Npy* and *Cck*, and *Vwa5a*, though detected at low levels in this dataset, may be an exclusive marker of Ivy cells.

It is commonly held that isocortical neurogliaform cells, unlike hippocampal cells, have an exclusively CGE origin. Nevertheless, the *Cacna2d1.Lhx6* classes are strongly homologous to an isocortical class "Igtp" described by Tasic et al [21], which expresses several markers we hypothesize to correspond to neurogliaform cells, as well as *Lhx6*. We therefore hypothesize that the isocortex also contains an MGE-derived neurogliaform population, that is too rare to have been detected in previous studies.

### *5. Cells expressing Cacna2d1 and Ndnf: CGE neurogliaform*

We identified continent 5 as CGE-derived neurogliaform cells. These cells shared many markers with the putative Ivy and MGE-neurogliaform cells on continent 4, including the neurogliaform-associated genes *Reln, Npy,* and *Gabrd* as well as novel markers *Cacna2d1 Cacna2d1, Lamp5, Cryab, Id2, Hapln1, Nfix,* and *Bcl11b*. However, they lacked *Lhx6* and expressed *Nos1* at most weakly, indicating CGE rather than MGE lineage [6]. Cells of this group were further distinguished from the putative Ivy/MGE-neurogliaform class by expression of *Kit* (which has been associated with CGE-derived neurogliaforms in isocortex; Miyoshi et al, SFN Abstract 208.06, 2014) and *Ndnf*, which has been suggested as a unique identifier of isocortical neurogliaforms cells by Tasic et al [21]. While our data certainly support expression of *Ndnf* in CGE-derived neurogliaform cells, we suggest that in CA1 *Ndnf* is neither necessary for identification of a cell as neurogliaform (as the putative Ivy/MGE-neurogliaform cells do not express it), nor sufficient (as some other classes also express *Ndnf*, to be discussed below).

The putative CGE-neurogliaform cells were split into to three clusters. The first two of these (*Cacna2d1.Ndnf.Npy* and *Cacna2d1.Ndnf.Rgs10*) showed a relationship that paralleled that of putative Ivy and MGE-neurogliaform cells. Indeed, *Cacna2d1.Ndnf.Npy* showed larger latent factor values (mean -0.04), together with stronger expression of *Cck, Npy, Caln1, Kcnip1, Gabrd, Slc6a1*, while *Cacna2d1.Ndnf.Rgs10* (mean latent factor -0.58) showed stronger expression of *Reln, Fxyd6* and *6330403K07Rik*. Thus, while these cells differ substantially from the putative MGE-derived neurogliaform and Ivy cells in group *Cacna2d1.Lhx6*, we speculate that they might show similar properties, and specifically that *Cacna2d1.Ndnf.Npy* might reflect a proximally-targeting CGE-derived homolog of the Ivy cell.

The third cluster of putative CGE-neurogliaform cells (*Cacna2d1.Ndnf.Cxcl14*) was characterized by expression of markers including *Rxfp1, Npas3* as well as *Cxcl14, Rgs12* (which likely localize these cells to the R-LM border; see below), but lacked *Pnoc, Rgs10*, and *Car4*. While this class was quite distinct at a transcriptional level (isolation metric 40.2 bits/cell), the molecular markers that distinguish them from other putative neurogliaform cells have not to our knowledge been examined in reconstructed cells, precluding identification of this subclass with the literature. Nevertheless, we note that the distinction between *Cacna2d1.Ndnf.Cxcl14* and the rest of *Cacna2d1.Ndnf* appears homologous to the distinction between the *Ndnf Cxcl14* and *Ndnf Car4* classes of putative isocortical neurogliaform cells defined by Tasic et al [21].

Finally, closely associated with this group were two small clusters that showed "hybrid" expression patterns. Cells of the group *Calb2.Cryab* (50 cells; isolation metric 38.6 bits/cell) showed a pattern intermediate between the *Cacna2d1.Ndnf.Cxcl14* cluster, and putative IS1 cells (described below). Cells of the group *Sst.Cryab* (27 cells; isolation metric 76.5 bits/cell) showed an unexpected

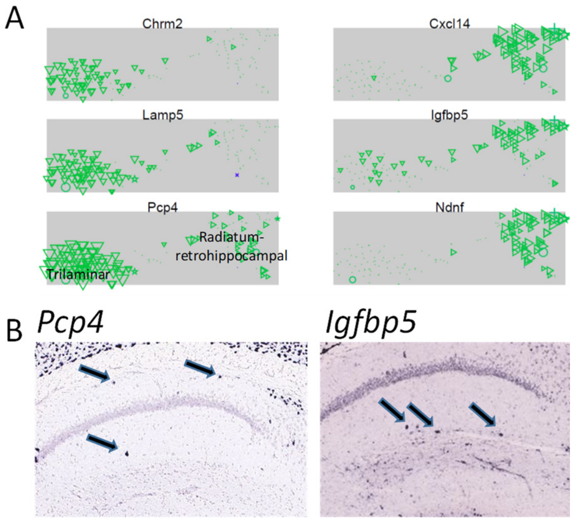


Fig **C6**. Two classes in continent 6 were identified as projection neurons. **A,** Cells in cluster *Ntng1.Chrm2* were identified as trilaminar cells by their strong expression of *Chrm2* but lack of *Sst* or *Pvalb;* they also strongly expressed *Pcp4*, but were not the only cells to do so. Cells in cluster *Ntng1.Rgs10* were identified as radiatum-retrohippocampal cells. They expressed primarily novel markers including *Igfbp5*, which localized them to the R-LM border. Symbols are as in main text Fig 2. **B,** Allen atlas images reveal a cohort of *Pcp4* cells in *so*, and *Igfbp5* at the *sr/slm* border.

expression pattern intermediate between CGE-neurogliaform cells, and hippocamposeptal cells. This strange group showed a split location on the expression map, with most located with the neurogliaform cells on continent 5, but six cells with high expression of *Sst* located off the shore of continent 1. This group might represent a rare novel cell type, but given the unexpected nature of this expression pattern further confirmation is required to confirm it does not result from an artifact such as cell merging.

### *6. Cells expressing Ntng1 and Rgs4:* CGE-derived *projection cells*

The hippocampus contains several types of long-range GABAergic projection neuron [42]. As described in previous sections, many of these express *Sst*, including hippocamposeptal cells and (we suggest) backprojection cells. However, at least two types of long-range GABAergic neurons do not express *Sst* [12,42,43]. Trilaminar cells project from *stratum oriens* to the subiculum but not the septum, and lack SST but express CHRM2. Radiatum-retrohippocampal cells project to retrosplenial cortex, are located at the border of strata *oriens* and *lacunosum-moleculare*, and lack most known molecular markers (they are negative for *Sst, Pvalb, Calb1, Calb2,* and *Cck*, and express *Chrm2* at most rarely); molecular markers positively identifying radiatum-retrohippocampal neurons have not yet been described.

The next group of cells we consider consists of two clusters located in the central west of the nbtSNE map, that we identify with putative long-range projection cells (**Fig C6**). Unlike *Sst*-positive projection cells, both lacked *Lhx6*, indicating a developmental origin in the CGE. We identified the first of these clusters (*Ntng1.Chrm2;* 74 cells) with trilaminar cells. Indeed, these cells express *Chrm2* more strongly than any other cells in the database, and lack *Sst* and *Pvalb.* They therefore likely correspond to the strongly CHRM2-positive cells identified at the *alveus/oriens* border lacking septal projections identified by Hajos et al [29], and to the SST-/CHRM2+ neurons identified by Jinno et al [12]. While we did not identify a molecular marker that uniquely identified this class, their molecular profile was highly unique (isolation metric 111.3 bits/cell), and they were characterized by strong expression of several genes including *Ntng1, Pcp4, Lamp5, Sema5a, Rgs4, Gda, Grin2a,* and *Bcl11b*; some of them also expressed *Grm1,* although more weakly than putative hippocamposeptal, O-LM or backprojection cells [c.f. Jinno et al [12]]. Interestingly, we note that *Pcp4* was also strongly expressed in the classes identified with backprojection and hippocampo-septal cells, suggesting that it

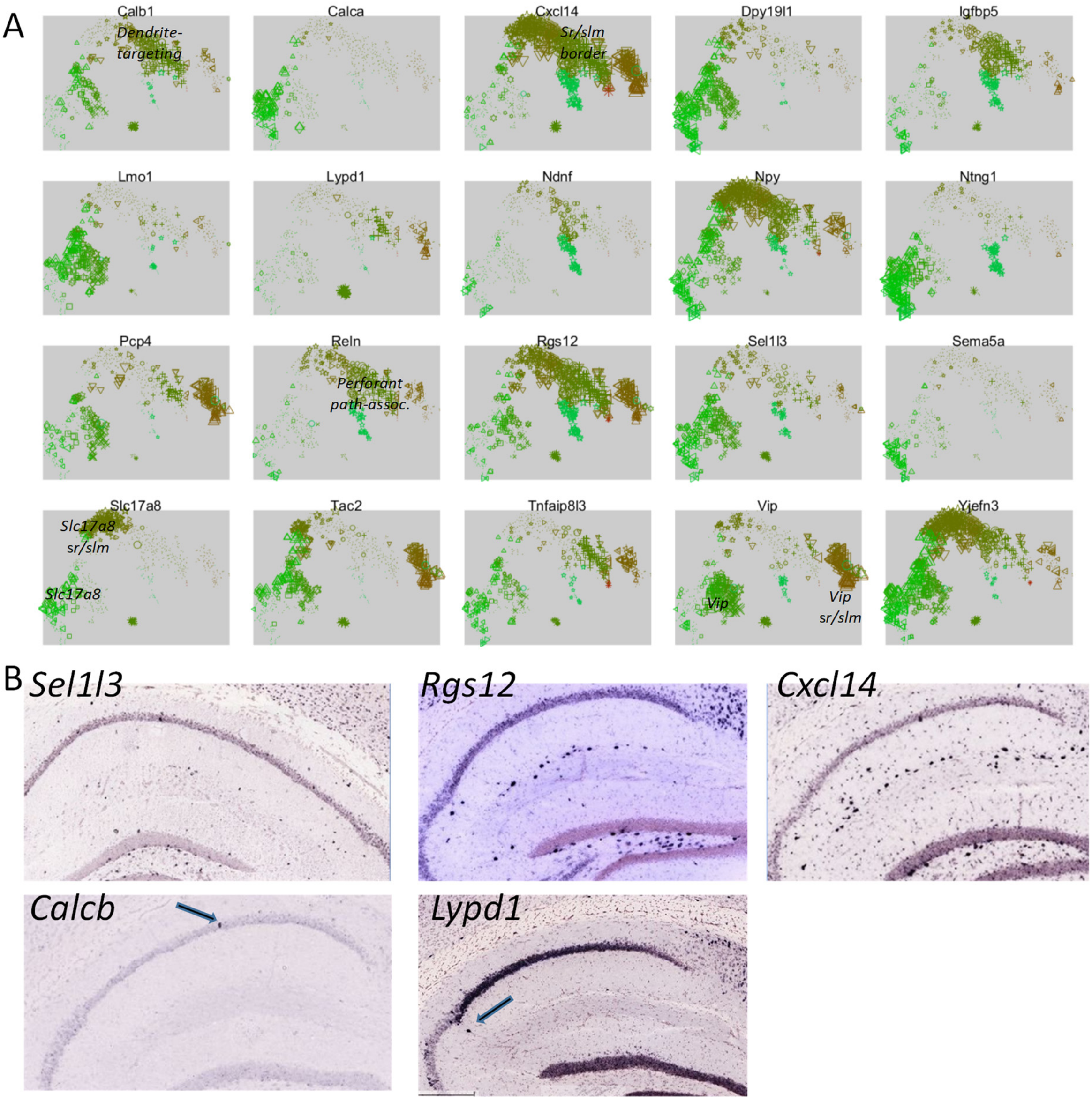


Fig **C7-8**. *Cck*-expressing interneurons formed a highly diverse group, spread over continents 7 and 8. All cells strongly expressed *Cck* and *Cnr1* (main text **Fig 2**). **A,** *Cck* subtypes were distinguished by expression of many genes. Cells on continent 7 expressed one or more of *Dpy19l1*, *Lmo1*, *Sel1l3*, while cells of continent 8 were distinguished expression of *Cxcl14* and *Rgs12.* A large island to the east of continent 8 strongly expresses both *Vip* and *Cxcl14.* Symbols are as in main text Fig 2. **B,** Allen atlas images reveal continent 7 cells can be found in any layer, while continent 8 cells are localized to the *sr/slm* border. Rare cell types were identified expressing calcitonin precursors (*Calca* and *Calcb*), and *Lypd1* which is localized close to the CA3 border.

may be an informative (but not exclusive) marker of long-range projection GABA cells.

The second cluster in this group (*Ntng1.Rgs10*; 72 cells; isolation metric 64.7 bits/cell), located to the east of *Ntng1.Chrm2*, was harder to identify but we hypothesize it may correspond to radiatum-retrohippocampal cells. These cells also expressed *Ntng1, Bcl11b, Gda, Rgs4, Grin2a* and *Pcp4* (although the latter more weakly than *Ntng1.Chrm2* cells), but lacked *Chrm2, Lamp5* and *Sema5a*, and expressed a number of additional markers including *Cxcl14, Igfbp5, Ndnf*, and *Reln*, which localize to

the radiaum/lacunosum-moleculare border (**Fig C6, C7-8**). This fact, combined with their general similarity to the *Ntng1.Chrm2* cluster and lack of markers absent from radiatum-retrohippocampal cells (*Sst, Pvalb, Calb1, Calb2, Cck, Chrm2*)[12,43], lead us to hypothesize they are radiatum-retrohippocampal cells.

Although *Ndnf* has been proposed as a unique marker of isocortical neurogliaform cells [21], we do not identify this class as neurogliaform cells due to their lack of *Gabrd* and several other neurogliaform markers. Instead, we suggest that at least in CA1, *Ndnf* is not exclusively found in neurogliaform cells.

### *7. Cells expressing Cck and Sel1l3: Cck cells in any layer*

The neuropeptide cholecystokinin (CCK) is expressed to some degree in many hippocampal inhibitory and excitatory neuronal classes. Nevertheless, CCK is found at exceptionally high levels in specific interneuron subtypes, which are often referred to simply as CCK interneurons [44–46]. We will follow this terminology here, with the understanding that other neurons also express *Cck* mRNA at lower levels. *Cck* interneurons are CGE-derived [7], and have several molecular characteristics including strong expression of the cannabinoid receptor CNR1 [47]; the absence of PVALB and SST [48]; the presence of muscarinic receptors CHRM1 and CHRM3 but not CHRM2 [44,49]; and synaptic release controlled primarily by N-type rather than P/Q type $Ca^{2+}$ channels (i.e. CACNA1B rather than CACNA1A) [50]. Physiologically, these cells are usually regular spiking, although some exhibit a fast-spiking phenotype [51].

*Cck* interneurons are extremely heterogeneous in their gene expression and morphology [51–55]. Their somata can be located in all layers, but are most common in *sp* and *sr*, and are particularly dense at the *sr*/*slm* border [51,55]. They exhibit many morphological subtypes: basket cells targeting pyramidal cell somata, but whose own somata need not lie in *sp*; apical dendrite-innervating cells targeting proximal apical dendrites; Schaffer collateral-associated cells, whose somata are located in *sr* and target pyramidal dendrites in the same layer; and perforant path-associated cells that target pyramidal tufts in *slm*. CCK-expressing cells can be divided into largely non-overlapping subtypes expressing VIP, CALB1, or SLC17A8 (the vesicular glutamate transporter, VGLUT3), [55,56], although some CCK cells express none of these three, and a small population expressing both SLC17A8 and CALB1 are found at the border with CA3 [55]. Data so far suggest that VIP/CCK co-expressing cells are primarily basket cells with somas in *sp* [56], SLC17A8-expressing cells are primarily basket cells whose somas need not be in *sp* [55], and that at least some dendrite-targeting CCK cells express CALB1 [52,53].

Our database contained a large number cells (n=818) that were confidently identified as *Cck* interneurons (**Fig C7-8**). Indeed, these cells all showed strong expression of *Cck* and *Cnr1;* lacked *Lhx6, Sst* and *Pvalb;* showed weak expression of *Cacna1a* but stronger expression of *Cacna1b;* expressed *Chrm1* and *Chrm3* more strongly than all other classes; and also expressed *Sncg*, which defines homologous classes of cells in isocortex [21]. An unexpected feature of these neurons was the sometimes strong expression of *Npy*, which has not yet been investigated in *Cck* cells in rat. Immunohistochemical analysis however confirmed that NPY was often – though not always – present in CCK interneurons (**Fig S7**).

As expected, these putative *Cck* interneurons were highly diverse, falling into 13 clusters which in many cases appeared lie along a continuum. These cells fell into two major

groups, respectively were located on expression map continents 7 and 8 (**Fig C7-8**). The cells of continent 7 were characterized by expression of *Dpy19l1, Sel1l3, Pbx1,* and/or *Serpini1,* whereas cells on continent 8 expressed *Cxcl14, Rgs12, Reln, Serpine2, Prkca, Syt6,* and/or *Cpne5*. Based on the laminar expression of these genes revealed by the Allen atlas (**Fig C7-8B**) we hypothesize that cells of continent 7 can lie in any layer, while those of continent 8 constitute a specialized subclass of CCK cell located at the *sr*/*slm* border.

The cells of continent 7 fell into six clusters. The first of these (*Cck.Sema5a*) showed some similarities with the putative long-range projection cells to their south, expressing *Ntng1, Sema5a, Bcl11b, Gda,* and *Grin2a*, but they differed from them not only by much stronger expression of *Cck* but also by expression of *Scrg1, Alcam, Kcnk2,* AND lack of *Pcp4* and *Rgs4.* Some of these cells, located at the far western coast, expressed *Npy.* A lack of previously-investigated markers means we are unable to confidently identify these neurons with any described in the literature, but speculate they might represent a class of neurons with both *Cck* expression and subicular projections. This cluster transitioned apparently continuously into a second cluster (*Cck.Calca,* n=31 cells; isolation metric 34.6 bits/cell) located at the western edge of continent 7, and uniquely characterized by expression of the Calcitonin gene-related peptide (CGRP) precursors *Calca* and *Calcb.* This cluster also showed expression of *Ntng1* but lacked other markers of putative projection cells including *Bcl11b, Sema5a,* and *Gda.* Immunohistochemistry in rat showed CGRP expression rarely in CA1, in scattered cells located close to subiculum [57]; however the Allen atlas indicates that in mouse *Calca* and *Calcb* can also be found in rare neurons located in *sp, so,* and proximal *sr* of CA1 proper (**Fig C7-8**). A subset of *Cck.Sema5a* and *Cck.Calca* cells expressed *Slc17a8, w*hile *Vip* and *Calb1* expression was weak in these cells. We therefore hypothesize that these cells represent *Vip*-negative, *Calb1*-negative *Cck* basket cells located close to the pyramidal layer.

The remaining four clusters of continent 7 all expressed the transcription factor *Lmo1*, a transcription factor expressed in hippocampal pyramidal cells and scattered interneurons in multiple layers [58]. These *Lmo1*-positive cells, located toward the east of the continent, were divided by a "river" that separated cells expressing *Vip* to the southeast, from cells expressing *Npy* to the northwest, while *Tac2*-expressing cells were found on both sides.

The *Vip*/*Cck* cell class closest to the "river" dividing *Vip-* from *Npy-* expressing cells (*Cck.Lmo1.Vip.Fam19a2;* n=58 cells) showed an expression pattern consistent with a fast-spiking phenotype. Compared to other *Cck* cells, this class showed stronger expression of fast-spiking associated genes (e.g. *Kcnc1, Kcna1, Syt1, Scn1a*); genes involved in presynaptic and vesicular function (e.g. *Cnr1, Gad1, Slc6a1, Syp, Sv2a, Cplx1*); neurofilaments (*Nefh, Nefl*) and a related intermediate filament protein *Krt73;* energy and ion rebalancing (*Atp1b1,* mitochondrial genes); GABA$_B$ receptor function (*Gabbr1* and *Kctd12,* which subserves desensitization of GABA$_B$ receptors in hippocampal Cck interneurons [59]), as well as other genes whose function is presently less clear (*Alcam, Rgs8, Col25a1, Trp53i11, Yjefn3*). Consistently, this cluster's mean latent factor was 1.45, comparable to that of *Pvalb*-positive neurons. We suggest that these cells correspond to the minority of *Cck* interneurons with a fast-spiking phenotype [51]. Furthest from the river was the class *Cck.Lmo1.Vip.Crh*, characterized by expression of the neuropeptide *Crh* as well as stronger

expression of *Vip* itself, together with many other genes associated with the putatively slower-spiking end of the latent factor continuum (e.g. *Fxyd6, 6330403K07Rik, Crim1, Nrsn1, Pcp4, Fxyd7, Egln3, Marcksl1, Nfix, Cryab, Zbtb20, Tpd52l1*). The third class of *Vip/Cck* cells (*Cck.Lmo1.Vip.Tac2*) located on the northestern shore of continent 7, occupied an multiple positions along this continuum, but was distinguished by its expression of the neurokinin-B precursor gene *Tac2*, which has been shown to be expressed in a subset of Cck interneurons [53]. Cells to the northwest of the river expressed *Npy* but lacked *Vip.* These neurons (*Cck.Lmo1.Npy*), contained a substantial population of cells expressing *Tac2*, occasionally *Calb1*, but rarely *Slc17a8*, and we suggest that they correspond primarily to pyramidal-layer *Cck* interneurons lacking in other previously-described markers. Interestingly, the expression of *Npy* and *Vip* was highly complementary even within the population of *Cck* interneurons, suggesting that these neuropeptides and corresponding cell populations might have antagonistic functions in controlling hippocampal circuitry.

#### 8. Cells expressing Cck and Cxcl14

We identified the second major group of *Cck* interneurons, located on continent 8, as a specialized set of *Cck* cells located at the *sr/slm* border. These cells express *Cxcl14, Rgs12, Cpne5, Prkca, Syt6* and/or *Reln (*several of which are localized at the *sr/slm* border, **Fig** C7-8*)*, but not *Dpy19l1*, *Sel1l3*, *Pbx1*, or *Serpini1* which instead characterize continent 7. Additional *in situ* hybridization analysis confirmed that *Cxcl14* is located at the *sr/slm* border, and overlaps with many key markers of this class (main text **Fig 8**). Cells of this group are again highly diverse, falling into seven clusters.

Four clusters of this group were arranged in an apparent continuum from west to east along continent 8 (*Cck.Cxcl14.Slc17a8, Cck.Cxcl14.Calb1.Kctd12, Cck.Cxcl14.Calb1.Igfbp5, Cck.Cxcl14.Calb1.Tnfaip8l3*), corresponding to a range of hidden factor values and likely reflecting continuous gradation of both spiking characteristics and axon targets, as discussed in main text (**Fig 6A,B**). *Npy* was expressed in the western half of the continent, identified with soma-targeting cells. *Calb1,* which identifies dendrite targeting cells [53,60] expressed in the center and east of the continent, while *Reln,* a marker of cells whose axons target *slm* [9] was found in the far east, leading to identify the cluster *Cck.Cxcl14.Calb1.Tnfaip8l3* with perforant-path associated cells. Expression of *Tnfaip8l3* in this latter class leads us to suggest that this molecule, at least in hippocampus, is not sufficient to identify neurogliaform cells, as had been previously speculated [61].

An additional cluster in this group (*Cck.Lypd1*) was small and highly distinct (n=17 cells, isolation metric 129.3 bits/cell), located on an isolated island far to the south of the main continent. These cells express *Cck* and *Cxcl14* but lack many other genes typical *Cck* interneurons, including *Sncg, Gap43, Snca,* while expressing several atypical genes including *Lypd1* and *Satb1.* Cells in this group express both *Calb1* and *Slc17a8*, a combination found in a rare class of neurons found in *sr* close to the border of CA1 and CA3 [55], where *Lypd1* expression can be occasionally observed (**Fig C7-8**). We therefore suggest that this cluster represents a rare class of *Cck* cell found in this location. An additional small cluster (*Cck.Cxcl14.Calb1.Tac2*; n=36 cells) was characterized by expression of *Tac2* (which is often collocated with *Cck* and *Calb1* in rat; [9]) as well as *Pcp4* and *Egln3.* These cells were located at multiple points of the expression map, indicating potential further subdivisions within this small class.

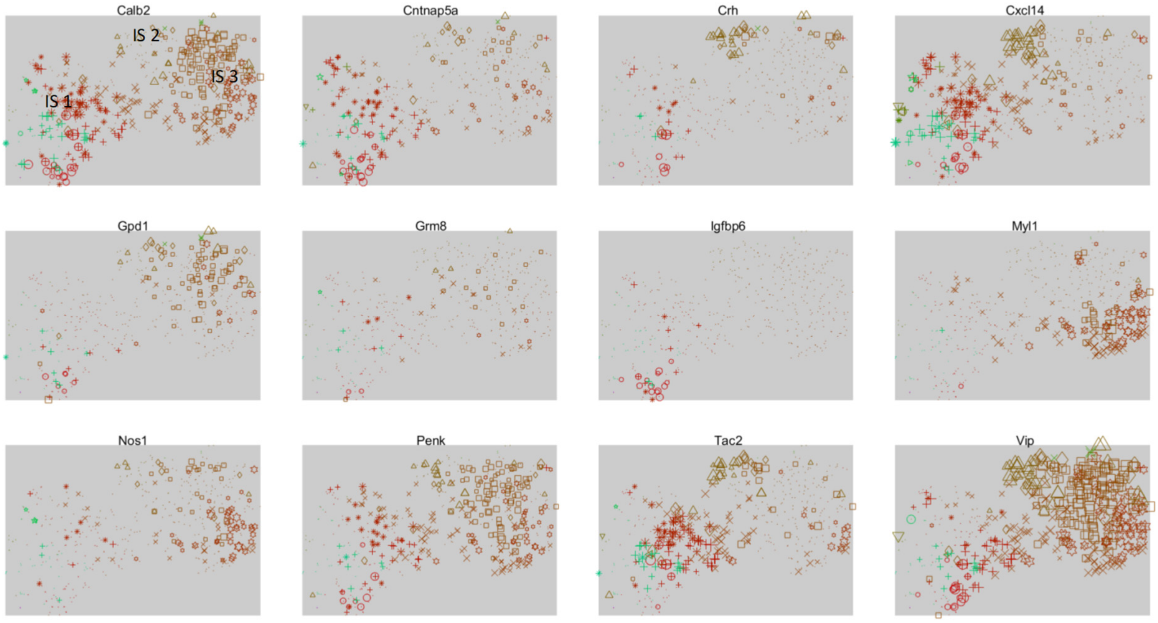


Fig **C9**. Neurons on continent 9 were identified as interneuron selective (I-S) cells. Cells on the western half of the continent, which expressed *Calb2* but of which only a minority expressed *Vip*, were identified as IS-1 cells. *Vip* was expressed on the eastern half, with a *Calb2*-negative group in the north identified as IS-2 and the *Calb2*+ south as IS-3. Cells at the south-eastern end of the continent formed a subtype of IS-3 cells expressing *Myl1* and *Nos1*. Symbols are as in main text Fig 2.

Expression of *Vip* was barely found on the mainland of continent 8, but *Vip* was strongly expressed by a distinct cluster of cells located on a large island to its east (*Cck.Cxcl14.Vip*; n=72 cells; isolation metric 56 bits/cell). These cells strongly expressed both *Vip* and *Cck*, and were differentiated from the *Vip*/*Cck* cells on continent 7 by their additional strong expression of *Cxcl14*. This group presented a puzzle, since *Cxcl14* is located primarily at the *sr*/*slm* border, whereas immunohistochemistry in rat has localized CCK/VIP basket cells to *sp* [56]. Because *Cxcl14* expression can be found in *sp* on rare occasions, we tested whether this cluster reflects *sp* cells, by combining in situ hybridization for *Cxcl14* with immunohistochemistry against VIP in mouse CA1 (main text, **Fig 10**). This revealed frequent co-expression at the *sr*/*slm* border (18/255 *Cxcl14* cells positive for VIP; 18/94 VIP cells positive for *Cxcl14*), but very few *Cxcl14* cells in *sp*, and essentially no double labelling (1 of 95 VIP cells was weakly labelled for *Cxcl14)*. It is possible that this discrepancy reflects a species difference between mouse and rat; however, Somogyi et al [55] found that small fraction (4.3 +/- 3.1%) of CCK cells in *sr* and *slm* do express VIP, potentially representing these *Cck.Cxcl14.Vip* cells. Cells in this cluster expressed many molecules typical of putative *Cck* basket cells (e.g. *Cnr1, Kctd12, Sncg, Cadps2*), and occasionally *Calb1* or *Reln*. We therefore speculate that they represent a class of dendrite-targeting *Vip*/*Cck* neuron located at the *sr*/*slm* border, that were either too rare to be detected in previous studies in rat, or not present in that species. Nevertheless, we also note that Acsady et al [56] found that a small fraction of VIP cells located at the *sr*/*slm* border and targeting stratum radiatum were immunopositive for *Cck* (6/72, 8%), which they identified as interneuron-selective. Confident

identification of this cluster therefore requires further work.

A final cluster of cells (*Ntng1.Synpr),* was located on a peninsula to the south continent 8. Unlike the main continuum of *Cck/Cxcl14* neurons, these cells expressed *Ntng1* and *Ndnf,* and in hierarchical clustering they appeared close to the *Ntng1.Rgs10* cluster of putative radiatum-retrohippocampal neurons (main text **Fig 2**). We note that these cells lacked *Sncg,* which was almost ubiquitous in putative *Cck* interneurons, and that only cells at the north of the peninsula expressed *Cck* itself. We are unable to identify this cluster with any group of cells previously identified in the literature and speculate that it might represent a novel class of *Cck*-positive projection interneurons.

### *9. Cells expressing Penk, Calb2 and/or Vip*: interneuron selective interneurons

A subset of hippocampal interneurons specifically inhibit other GABAergic cells. These interneuron selective (I-S) cells are characterized by expression of PENK, CALB2 and/or VIP, and can be divided into at least three subclasses by their morphology and neurochemical content [56,62–64]. The first class (IS-1) are located in all layers, have axons primarily in *sr* coursing in all directions, and exhibit "dendritic braids" likely representing gap-junction coupling. IS-1 cells are immunopositive for CALB2, but not necessarily for VIP. A second class, termed IS-2 cells express VIP but not CALB2 and have small somata, dendrites restricted to *slm*, and an axonal arbor that descends vertically through *sr*. A related class, considered to be a subtype of IS-2 cell, have similar axonal arbor but dendrites spanning all layers; these cells express VIP, while half also express CALB2 and a small fraction express CCK. Finally, IS-3 cells have somata in *sp* or *sr* and an axon that targeting *so*. These cells express both VIP and CALB2. Many interneuron-selective cells also express PENK [65,66].

Continent 9 contained a diverse collection of cells (n=482 cells, 8 clusters) expressing *Penk* together with either or both of *Calb2* and *Vip*, which we therefore identified as I-S cells. Continent 9 was split into an eastern half identified with IS-2 and IS-3 cells, and a western half associated with IS-1 cells (**Fig C9**). Cells of continent 9 had the lowest values of the latent factor of the entire database, suggesting slow spiking, weak GABA release, and low metabolism. They expressed mitochondrial genes the most weakly of all cells in the database, consistent with anatomical observations [67]. Total RNA expression levels were also lower in these cells compared to the database as a whole, consistent with the small size of I-S cells.

As with previous cell groups, these clusters often appeared to run into each other as a continuum. However, whereas previously described cell groups tended to show similar correlation patterns (main text **Fig 6**), correlations amongst the putative I-S cells were largely unrelated to those of previous cell groups, even when the same genes were expressed. For example, *Fxyd6* and *Atp1b1* were positively correlated in this group (not shown).

The majority of cells in the eastern half of continent 9 expressed both *Calb2* and *Vip*, and were therefore identified as IS-3 cells. These cells fell into three clusters representing an apparent continuum of gene expression. Cells in the northeast (*Calb2.Vip.Gpd1* n=121 cells) more strongly expressed a largely novel set of genes including *Gpd1, Crip2, Igsf3, Nfix, Vwc2, Ccnd2, Trp53i11, Cpne7, Pthlh, Zcchc12;* a subset of them expressed *Grm8,* a metabotropic glutamate receptor that has been identified in VIP-positive terminals targeting trilaminar

cells [68]. Cells further to the south (cluster *Calb2.Vip.Nos1,* n=60 cells) more strongly expressed *Myl1, Nos1, Alcam, Nacc2, Rgs12.* We note that *Nos1* has previously been reported in a subset of IS-cells [6], and that many of the genes typical of this second group are found in the "Vip Mybpc2" class defined by Tasic et al [21], which may therefore be homologous. On the southern tip of this group were cells that did not express *Nos1* but instead contained *Cox6a2, Lypd1,* and *Serpine2*, and constituted part of a heterogeneous cluster (*Calb2.Vip.Igfbp4*, n=101 cells) that also extended to the western island. We were not able to identify these three clusters with subclasses of IS-3 cell described in the literature, and speculate that this continuum might represent modulation of IS-3 gene expression by activity.

An additional cluster in the eastern half (*Vip.Crh.Pcp4,* n=58 cells) expressed *Vip* but expressed *Calb2* at most weakly, and was therefore identified as representing IS-2 neurons. These cells also expressed *Cxcl14,* consistent with the location of IS-2 cells at the *sr*/*slm* border [56], and also expressed the neuropeptides *Crh* and *Tac2.* This cluster was spread over two locations in the nbtSNE map, with a subset of *Cck*-expressing cells located on an island close to the *Cck.Cxcl14.Vip* cluster (**Fig C7-8**), and subset lacking *Cck* located at the northwestern corner of the eastern island of continent 9; however, the ProMMT algorithm did not split this cluster as difference between their expression patterns was insufficient for the BIC criterion. We speculate that these two subclusters might correspond to the two dendritic subtypes of IS-2 cell, which differ in *Cck* expression [56]. A small, related cluster (*Vip.Crh.C1ql1,* n=18 cells) was similar to the *Cck*-negative half of *Vip.Crh.Pcp4,* but lacked expression of *Pcp4* while instead expressing *Zbtb20* and *C1ql1.*

We identified cells on the western half of continent 9 with IS-1 cells, as many of them contained *Calb2* but not *Vip*. In addition to *Vip*, these neurons were less likely to express *Gabra1, Cit, Caln1, Pthlh, Rgs10, Limch1, Sparcl1, Sez6, Maf,* but more likely to express *Cntnap5a, Cadps2, Bcl11a, Parm1, Sstr1, Pcdh9, Qpct, Clstn2, Tac2, Cxcl14, Rgs12.* These cells showed considerable diversity. A group of cells in the center and north of the island (*Calb2.Cntnap5a.Rspo3*) showed relatively strong expression of genes including *Rspo3, Tac2, Igfbp4, Trhde, Cbln2, Clstn, Syt1, 2610001J05Rik, Cxcl14* and *Rgs12,* which likely indicates a location at the R-LM border. This cluster merged continuously into the previously described cluster *Calb2.Vip.Igfbp4,* which appeared to connect the eastern and western islands. A cluster to the southwest (*Calb2.Cntnap5a.Igfbp6,* n=25 cells) was characterized by *Igfbp6, Nnat, Ntng1, Epha5,* and absence of *Mef2c.* A group of cells along the southeastern coast (*Calb2.Cntnap5a.Vip,* n=51 cells) expressed *Vip* as well as *Calb2*, but were distinguished from the putative IS-3 cells on the eastern island by their expression of *Cntnap5a, Nnat, Tac2*, lack of *Caln1, Cit, Gabra1,* as well as weaker expression of *Vip* itself. A final cluster (*Calb2.Cryab*) joined these putative IS-1 cells to the *Cxcl14*-expressing putative neurogliaform cells to their west, and indeed expressed an apparent hybrid pattern between putative IS-1 and neurogliaform genes (*Calb2, Cntnap5a, Reln, Id2, Cryab*).

Anatomically reconstructed IS-1 cells are diverse: despite their common *Calb2* expression and axonal arborization in *sr*, these cells may have somas in any layer, and it has not been demonstrated that they all lack VIP [63,64]. We speculate that the diverse clusters in the western half of continent 9 represent IS-1 cells with somata in different layers, and further speculate that there exist a small

number of cells corresponding to the *Calb2.Cryab* cluster showing hybrid characteristics of IS-1 and neurogliaform cells.

### *10. Cells expressing Sst, Nos1, and Penk*

A small number of cells (n=35) constituted an exceptionally distinct cluster termed *Sst.Nos1,* which was exhibited by far the greatest *Nos1* expression in the database. In addition, the *Sst.Nos1* cluster was characterized by a unique gene combination including *Sst*, *Grm1, Chrm2, Penk, Pcp4, Npy*, *Oxtr, Ntn1* as well as some genes they exclusively expressed amongst CA1 interneurons such as *Chodl*. This expression profile was strikingly similar to an isocortical group detected in previous transcriptomic studies, termed Int1 [69] or "Sst Chodl" [21], that has been identified with sleep-active GABAergic long-range projection neurons [70,71]. Their unique profile resulted in a very high isolation metric (227.9 bits/cell), and an "island" location on the expression map, far to the north-east of the *Sst/Grm1* mainland (shown as an inset in **Fig C1**).

Although *Sst/Nos1* co-expression is rarer in hippocampus than in isocortex, rare neurons strongly co-expressing SST and NOS1 have been detected immunohistochemically in mouse CA1 [72]. These large neurons are intensely NOS1-immunopositive throughout the dendritic tree and also in their axons, and contrast with a larger population that express NOS1 at moderate level in the somata, proximal dendrites and axons, many of which express calcium binding proteins in different proportion [3].

Quantitative studies of testing NOS1, SST and NPY simultaneously in the CA1 have not been reported in the mouse to our knowledge, and in the rat hippocampus [9,38], triple immunopositive cells are a minor population (0.4 - 0.6%) of all NOS1-positive cells tested (n= 240; n= 592).These rare intensely NOS1-positive cells are distributed in all layers except in the *slm*, and are more frequent near the subiculum CA1 border. Here, we demonstrated their immunoreactivity for NPY, CHRM2, GRM1, PCP4 and PENK, as predicted for the *Sst1.Nos1* cluster (main text **Fig 7**).

We suggest that two previously reported GABAergic classes – that have previously been considered distinct from the class of large SST/NOS1 cells – also correspond to the *Sst.Nos1* cluster. The first of these are "backprojection cells", demonstrated from in vivo labeling and visualization of strongly NOS1-positive axons crossing the hippocampal fissure to CA3 and the dentate gyrus, against the main direction of hippocampal information flow [73,74]. The suggestion that *Sst.Nos1* cells' axons can cross the hippocampal fissure is further supported by observation of CHRM2+ axons and SST+ axons have been observed crossing the hippocampal fissure in rat hippocampus, bearing "drumstick-like" appendages [13,29] similar to those of backprojection cells (Sik et al., 1994).

The second additional class that we propose corresponds to the *Sst.Nos1* are PENK-positive neurons with projections to subiculum [66]. While tests for SST and NOS1 were not conducted in the originally reports of these neurons, those tests that were performed were consistent with the *Sst.Nos1* class, for example expression of GRM1 in the somatodendritic plasma membrane, and a lack of I-S cell markers such as VIP or CALB2 expression in dendrites. The current database shows no evidence of further subdivisions *of Sst.Nos1* cells, although this cannot be definitively ruled out without a larger sample. We therefore suggest that *Sst.Nos1* cells constitute a rare but highly distinct class of hippocampal inhibitory cell, with diverse long-range projections.

## REFERENCES


1. Matyas F, Freund TF, Gulyas AI. Immunocytochemically defined interneuron populations in the hippocampus of mouse strains used in transgenic technology. Hippocampus. 2004;14: 460–81. doi:10.1002/hipo.100191

2. Chittajallu R, Craig MT, McFarland A, Yuan X, Gerfen S, Tricoire L, et al. Dual origins of functionally distinct O-LM interneurons revealed by differential 5-HT(3A)R expression. Nat Neurosci. 2013;16: 1598–607. doi:10.1038/nn.3538

3. Jinno S, Kosaka T. Patterns of expression of calcium binding proteins and neuronal nitric oxide synthase in different populations of hippocampal GABAergic neurons in mice. J Comp Neurol. 2002;449: 1–25. doi:10.1002/cne.10251

4. Jinno S, Kosaka T. Patterns of expression of neuropeptides in GABAergic nonprincipal neurons in the mouse hippocampus: Quantitative analysis with optical disector. J Comp Neurol. 2003;461: 333–349. doi:10.1002/cne.10700

5. Jinno S, Kosaka T. Cellular architecture of the mouse hippocampus: a quantitative aspect of chemically defined GABAergic neurons with stereology. Neurosci Res. 2006;56: 229–45. doi:10.1016/j.neures.2006.07.007

6. Tricoire L, Pelkey KA, Daw MI, Sousa VH, Miyoshi G, Jeffries B, et al. Common origins of hippocampal Ivy and nitric oxide synthase expressing neurogliaform cells. J Neurosci. 2010;30: 2165–76. doi:10.1523/JNEUROSCI.5123-09.2010

7. Tricoire L, Pelkey KA, Erkkila BE, Jeffries BW, Yuan X, McBain CJ. A blueprint for the spatiotemporal origins of mouse hippocampal interneuron diversity. J Neurosci. 2011;31: 10948–70. doi:10.1523/JNEUROSCI.0323-11.2011

8. Lein ES, Hawrylycz MJ, Ao N, Ayres M, Bensinger A, Bernard A, et al. Genome-wide atlas of gene expression in the adult mouse brain. Nature. 2007;445: 168–76.

9. Fuentealba P, Klausberger T, Karayannis T, Suen WY, Huck J, Tomioka R, et al. Expression of COUP-TFII nuclear receptor in restricted GABAergic neuronal populations in the adult rat hippocampus. J Neurosci. 2010;30: 1595–609. doi:10.1523/JNEUROSCI.4199-09.2010

10. Baude A, Nusser Z, Roberts JDB, Mulvihill E, Jeffrey Mcllhinney RA, Somogyi P. The metabotropic glutamate receptor (mGluRl$\alpha$) is concentrated at perisynaptic membrane of neuronal subpopulations as detected by immunogold reaction. Neuron. 1993;11: 771–787. doi:10.1016/0896-6273(93)90086-7

11. Ferraguti F, Cobden P, Pollard M, Cope D, Shigemoto R, Watanabe M, et al. Immunolocalization of metabotropic glutamate receptor 1alpha (mGluR1alpha) in distinct classes of interneuron in the CA1 region of the rat hippocampus. Hippocampus. 2004;14: 193–215. doi:10.1002/hipo.10163

12. Jinno S, Klausberger T, Marton LF, Dalezios Y, Roberts JD, Fuentealba P, et al. Neuronal diversity in GABAergic long-range projections from the hippocampus. J Neurosci. 2007;27: 8790–804. doi:10.1523/JNEUROSCI.1847-07.2007

13. Katona I, Acsády L, Freund TF. Postsynaptic targets of somatostatin-immunoreactive interneurons in the rat hippocampus. Neuroscience. 1999;88: 37–55. doi:10.1016/S0306-4522(98)00302-9

14. McBain CJ, DiChiara TJ, Kauer JA. Activation of metabotropic glutamate receptors differentially affects two classes of hippocampal interneurons and potentiates excitatory synaptic transmission. J Neurosci Off J Soc Neurosci. 1994;14: 4433–4445.

15. Sylwestrak EL, Ghosh A. Elfn1 regulates target-specific release probability at CA1-interneuron synapses. Science. 2012;338: 536–40. doi:10.1126/science.1222482

16. Katona L, Lapray D, Viney TJ, Oulhaj A, Borhegyi Z, Micklem BR, et al. Sleep and movement differentiates actions of two types of somatostatin-expressing GABAergic interneuron in rat hippocampus. Neuron. 2014;82: 872–86. doi:10.1016/j.neuron.2014.04.007

17. Gulyás AI, Hájos N, Katona I, Freund TF. Interneurons are the local targets of hippocampal inhibitory cells which project to the medial septum. Eur J Neurosci. 2003;17: 1861–1872.

18. Takács VT, Freund TF, Gulyás AI. Types and synaptic connections of hippocampal inhibitory neurons reciprocally connected with the medial septum. Eur J Neurosci. 2008;28: 148–164. doi:10.1111/j.1460-9568.2008.06319.x

19. Tóth K, Freund TF. Calbindin D28k-containing nonpyramidal cells in the rat hippocampus: their immunoreactivity for GABA and projection to the medial septum. Neuroscience. 1992;49: 793–805.

20. Acsády L, Pascual M, Rocamora N, Soriano E, Freund TF. Nerve growth factor but not neurotrophin-3 is synthesized by hippocampal GABAergic neurons that project to the medial septum. Neuroscience. 2000;98: 23–31.

21. Tasic B, Menon V, Nguyen TN, Kim TK, Jarsky T, Yao Z, et al. Adult mouse cortical cell taxonomy revealed by

single cell transcriptomics. Nat Neurosci. 2016;19: 335–346. doi:10.1038/nn.4216

22. Xu X, Roby KD, Callaway EM. Mouse cortical inhibitory neuron type that coexpresses somatostatin and calretinin. J Comp Neurol. 2006;499: 144–160. doi:10.1002/cne.21101

23. Katona L, Micklem B, Borhegyi Z, Swiejkowski DA, Valenti O, Viney TJ, et al. Behavior-dependent activity patterns of GABAergic long-range projecting neurons in the rat hippocampus. Hippocampus. 2017;27: 359–377. doi:10.1002/hipo.22696

24. Buhl EH, Halasy K, Somogyi P. Diverse sources of hippocampal unitary inhibitory postsynaptic potentials and the number of synaptic release sites [see comments] [published erratum appears in Nature 1997 May 1;387(6628):106]. Nature. 1994;368: 823–8.

25. Hu H, Gan J, Jonas P. Interneurons. Fast-spiking, parvalbumin+ GABAergic interneurons: from cellular design to microcircuit function. Science. 2014;345: 1255263. doi:10.1126/science.1255263

26. Viney TJ, Lasztoczi B, Katona L, Crump MG, Tukker JJ, Klausberger T, et al. Network state-dependent inhibition of identified hippocampal CA3 axo-axonic cells in vivo. Nat Neurosci. 2013;16: 1802–1811. doi:10.1038/nn.3550

27. Dehorter N, Ciceri G, Bartolini G, Lim L, del Pino I, Marin O. Tuning of fast-spiking interneuron properties by an activity-dependent transcriptional switch. Science. 2015;349: 1216–20. doi:10.1126/science.aab3415

28. Donato F, Rompani SB, Caroni P. Parvalbumin-expressing basket-cell network plasticity induced by experience regulates adult learning. Nature. 2013;504: 272–6. doi:10.1038/nature12866

29. Hajos N, Papp EC, Acsady L, Levey AI, Freund TF. Distinct interneuron types express m2 muscarinic receptor immunoreactivity on their dendrites or axon terminals in the hippocampus. Neuroscience. 1998;82: 355–76.

30. Takahashi T. Postsynaptic receptor mechanisms underlying developmental speeding of synaptic transmission. Neurosci Res. 2005;53: 229–240. doi:10.1016/j.neures.2005.07.005

31. Losonczy A, Zhang L, Shigemoto R, Somogyi P, Nusser Z. Cell type dependence and variability in the short-term plasticity of EPSCs in identified mouse hippocampal interneurones. J Physiol. 2002;542: 193–210.

32. Borhegyi Z, Leranth C. Substance P innervation of the rat hippocampal formation. J Comp Neurol. 1997;384: 41–58.

33. Vruwink M, Schmidt HH, Weinberg RJ, Burette A. Substance P and nitric oxide signaling in cerebral cortex: anatomical evidence for reciprocal signaling between two classes of interneurons. J Comp Neurol. 2001;441: 288–301.

34. Kakegawa W, Mitakidis N, Miura E, Abe M, Matsuda K, Takeo YH, et al. Anterograde C1ql1 signaling is required in order to determine and maintain a single-winner climbing fiber in the mouse cerebellum. Neuron. 2015;85: 316–329. doi:10.1016/j.neuron.2014.12.020

35. Varga C, Oijala M, Lish J, Szabo GG, Bezaire M, Marchionni I, et al. Functional fission of parvalbumin interneuron classes during fast network events. Elife. 2014;3. doi:10.7554/eLife.04006

36. Pascual M, Rocamora N, Acsády L, Freund TF, Soriano E. Expression of nerve growth factor and neurotrophin-3 mRNAs in hippocampal interneurons: morphological characterization, levels of expression, and colocalization of nerve growth factor and neurotrophin-3. J Comp Neurol. 1998;395: 73–90.

37. Armstrong C, Krook-Magnuson E, Soltesz I. Neurogliaform and Ivy Cells: A Major Family of nNOS Expressing GABAergic Neurons. Front Neural Circuits. 2012;6. doi:10.3389/fncir.2012.00023

38. Fuentealba P, Begum R, Capogna M, Jinno S, Marton LF, Csicsvari J, et al. Ivy cells: a population of nitric-oxide-producing, slow-spiking GABAergic neurons and their involvement in hippocampal network activity. Neuron. 2008;57: 917–29. doi:10.1016/j.neuron.2008.01.034

39. Price CJ, Cauli B, Kovacs ER, Kulik A, Lambolez B, Shigemoto R, et al. Neurogliaform neurons form a novel inhibitory network in the hippocampal CA1 area. J Neurosci Off J Soc Neurosci. 2005;25: 6775–6786. doi:10.1523/JNEUROSCI.1135-05.2005

40. Olah S, Fule M, Komlosi G, Varga C, Baldi R, Barzo P, et al. Regulation of cortical microcircuits by unitary GABA-mediated volume transmission. Nature. 2009;461: 1278–81. doi:10.1038/nature08503

41. Dolphin AC. The $\alpha 2\delta$ subunits of voltage-gated calcium channels. Biochim Biophys Acta. 2013;1828: 1541–1549. doi:10.1016/j.bbamem.2012.11.019

42. Jinno S. Structural organization of long-range GABAergic projection system of the hippocampus. Front Neuroanat. 2009;3: 13. doi:10.3389/neuro.05.013.2009

43. Miyashita T, Rockland KS. GABAergic projections from the hippocampus to the retrosplenial cortex in the rat. Eur J Neurosci. 2007;26: 1193–204. doi:10.1111/j.1460-9568.2007.05745.x

44. Armstrong C, Soltesz I. Basket cell dichotomy in microcircuit function. J Physiol. 2012;590: 683–694. doi:10.1113/jphysiol.2011.223669

45. Freund TF. Interneuron Diversity series: Rhythm and mood in perisomatic inhibition. Trends Neurosci. 2003;26: 489–495. doi:10.1016/S0166-2236(03)00227-3

46. Freund TF, Katona I. Perisomatic Inhibition. Neuron. 2007;56: 33–42. doi:10.1016/j.neuron.2007.09.012

47. Katona I, Sperlagh B, Sik A, Kafalvi A, Vizi ES, Mackie K, et al. Presynaptically located CB1 cannabinoid receptors regulate GABA release from axon terminals of specific hippocampal interneurons. J Neurosci. 1999;19: 4544–58.

48. Somogyi P, Hodgson AJ, Smith AD, Nunzi MG, Gorio A, Wu JY. Different populations of GABAergic neurons in the visual cortex and hippocampus of cat contain somatostatin- or cholecystokinin- immunoreactive material. J Neurosci. 1984;4: 2590–2603.

49. Cea-del Rio CA, Lawrence JJ, Tricoire L, Erdelyi F, Szabo G, McBain CJ. M3 muscarinic acetylcholine receptor expression confers differential cholinergic modulation to neurochemically distinct hippocampal basket cell subtypes. J Neurosci. 2010;30: 6011–24. doi:10.1523/JNEUROSCI.5040-09.2010

50. Wilson RI, Kunos G, Nicoll RA. Presynaptic Specificity of Endocannabinoid Signaling in the Hippocampus. Neuron. 2001;31: 453–462. doi:10.1016/S0896-6273(01)00372-5

51. Pawelzik H, Hughes DI, Thomson AM. Physiological and morphological diversity of immunocytochemically defined parvalbumin- and cholecystokinin-positive interneurones in CA1 of the adult rat hippocampus. J Comp Neurol. 2002;443: 346–367. doi:10.1002/cne.10118

52. Cope DW, Maccaferri G, Marton LF, Roberts JD, Cobden PM, Somogyi P. Cholecystokinin-immunopositive basket and Schaffer collateral-associated interneurones target different domains of pyramidal cells in the CA1 area of the rat hippocampus. Neuroscience. 2002;109: 63–80.

53. Klausberger T, Marton LF, O'Neill J, Huck JH, Dalezios Y, Fuentealba P, et al. Complementary roles of cholecystokinin- and parvalbumin-expressing GABAergic neurons in hippocampal network oscillations. J Neurosci. 2005;25: 9782–93. doi:10.1523/JNEUROSCI.3269-05.2005

54. Mátyás F, Freund TF, Gulyás AI. Convergence of excitatory and inhibitory inputs onto CCK-containing basket cells in the CA1 area of the rat hippocampus. Eur J Neurosci. 2004;19: 1243–1256. doi:10.1111/j.1460-9568.2004.03225.x

55. Somogyi J, Baude A, Omori Y, Shimizu H, El Mestikawy S, Fukaya M, et al. GABAergic basket cells expressing cholecystokinin contain vesicular glutamate transporter type 3 (VGLUT3) in their synaptic terminals in hippocampus and isocortex of the rat. Eur J Neurosci. 2004;19: 552–69.

56. Acsady L, Arabadzisz D, Freund TF. Correlated morphological and neurochemical features identify different subsets of vasoactive intestinal polypeptide-immunoreactive interneurons in rat hippocampus. Neuroscience. 1996;73: 299–315.

57. Freund TF, Hájos N, Acsády L, Görcs TJ, Katona I. Mossy cells of the rat dentate gyrus are immunoreactive for calcitonin gene-related peptide (CGRP). Eur J Neurosci. 1997;9: 1815–1830.

58. Hinks GL, Shah B, French SJ, Campos LS, Staley K, Hughes J, et al. Expression of LIM protein genes Lmo1, Lmo2, and Lmo3 in adult mouse hippocampus and other forebrain regions: differential regulation by seizure activity. J Neurosci Off J Soc Neurosci. 1997;17: 5549–5559.

59. Booker SA, Althof D, Gross A, Loreth D, Müller J, Unger A, et al. KCTD12 Auxiliary Proteins Modulate Kinetics of GABAB Receptor-Mediated Inhibition in Cholecystokinin-Containing Interneurons. Cereb Cortex N Y N 1991. 2016; doi:10.1093/cercor/bhw090

60. Gulyás AI, Freund TF. Pyramidal cell dendrites are the primary targets of calbindin D28k-immunoreactive interneurons in the hippocampus. Hippocampus. 1996;6: 525–534. doi:10.1002/(SICI)1098-1063(1996)6:5<525::AID-HIPO5>3.0.CO;2-H

61. Pfeffer CK, Xue M, He M, Huang ZJ, Scanziani M. Inhibition of inhibition in visual cortex: the logic of connections between molecularly distinct interneurons. Nat Neurosci. 2013;16: 1068–76. doi:10.1038/nn.3446

62. Acsady L, Gorcs TJ, Freund TF. Different populations of vasoactive intestinal polypeptide-immunoreactive interneurons are specialized to control pyramidal cells or interneurons in the hippocampus. Neuroscience. 1996;73: 317–34.

63. Freund TF, Buzsaki G. Interneurons of the hippocampus. Hippocampus. 1996;6: 347–470.

64. Gulyás AI, Hájos N, Freund TF. Interneurons containing calretinin are specialized to control other interneurons in the rat hippocampus. J Neurosci Off J Soc Neurosci. 1996;16: 3397–3411.

65. Blasco-Ibanez JM, Martinez-Guijarro FJ, Freund TF. Enkephalin-containing interneurons are specialized to innervate other interneurons in the hippocampal CA1 region of the rat and guinea-pig. Eur J Neurosci. 1998;10: 1784–95.

66. Fuentealba P, Tomioka R, Dalezios Y, Marton LF, Studer M, Rockland K, et al. Rhythmically active enkephalin-expressing GABAergic cells in the CA1 area of the hippocampus project to the subiculum and preferentially innervate interneurons. J Neurosci. 2008;28: 10017–22. doi:10.1523/JNEUROSCI.2052-08.2008

67. Gulyás AI, Buzsáki G, Freund TF, Hirase H. Populations of hippocampal inhibitory neurons express different levels of cytochrome c. Eur J Neurosci. 2006;23: 2581–2594. doi:10.1111/j.1460-9568.2006.04814.x

68. Ferraguti F, Klausberger T, Cobden P, Baude A, Roberts JD, Szucs P, et al. Metabotropic glutamate receptor 8-expressing nerve terminals target subsets of GABAergic neurons in the hippocampus. J Neurosci. 2005;25: 10520–36. doi:10.1523/JNEUROSCI.2547-05.2005

69. Zeisel A, Munoz-Manchado AB, Codeluppi S, Lonnerberg P, La Manno G, Jureus A, et al. Brain structure. Cell types in the mouse cortex and hippocampus revealed by single-cell RNA-seq. Science. 2015;347: 1138–42. doi:10.1126/science.aaa1934

70. Dittrich L, Heiss JE, Warrier DR, Perez XA, Quik M, Kilduff TS. Cortical nNOS neurons co-express the NK1 receptor and are depolarized by Substance P in multiple mammalian species. Front Neural Circuits. 2012;6: 31. doi:10.3389/fncir.2012.00031

71. Gerashchenko D, Wisor JP, Burns D, Reh RK, Shiromani PJ, Sakurai T, et al. Identification of a population of sleep-active cerebral cortex neurons. Proc Natl Acad Sci U S A. 2008;105: 10227–10232. doi:10.1073/pnas.0803125105

72. Jinno S, Kosaka T. Patterns of colocalization of neuronal nitric oxide synthase and somatostatin-like immunoreactivity in the mouse hippocampus: quantitative analysis with optical disector. Neuroscience. 2004;124: 797–808. doi:10.1016/j.neuroscience.2004.01.027

73. Sik A, Ylinen A, Penttonen M, Buzsaki G. Inhibitory CA1-CA3-hilar region feedback in the hippocampus. Science. 1994;265: 1722–4.

74. Sik A, Penttonen M, Ylinen A, Buzsaki G. Hippocampal CA1 interneurons: an in vivo intracellular labeling study. J Neurosci. 1995;15: 6651–65.
